# Association between missense variants of uncertain significance in the *CHEK2* gene and hereditary breast cancer: a cosegregation and bioinformatics analysis

**DOI:** 10.3389/fgene.2023.1274108

**Published:** 2024-02-27

**Authors:** Natalia Alonso, Sebastián Menao, Rodrigo Lastra, María Arruebo, María P. Bueso, Esther Pérez, M. Laura Murillo, María Álvarez, Alba Alonso, Soraya Rebollar, Mara Cruellas, Dolores Arribas, Mónica Ramos, Dolores Isla, Juan José Galano-Frutos, Helena García-Cebollada, Javier Sancho, Raquel Andrés

**Affiliations:** ^1^ Aragon Health Research Institute (IIS Aragón), Zaragoza, Spain; ^2^ Medical Oncology Department, Hospital San Pedro, Logroño, Spain; ^3^ Biochemistry Department, University Hospital Lozano Blesa, Zaragoza, Spain; ^4^ Medical Oncology Department, University Hospital Lozano Blesa, Zaragoza, Spain; ^5^ Breast Unit, University Hospital Lozano Blesa, Zaragoza, Spain; ^6^ Biochemistry Department, University Hospital Arnau de Vilanova, Lleida, Spain; ^7^ Medical Oncology Department, University Hospital of Valld’Hebron, and Valld’Hebron Institute of Oncology, Barcelona, Spain; ^8^ General Surgery Department, University Hospital Lozano Blesa, Zaragoza, Spain; ^9^ Department of Biochemistry, Molecular and Cell Biology, Faculty of Science, University of Zaragoza, Zaragoza, Spain; ^10^ Biocomputation and Complex Systems Physics Institute (BIFI), Joint Units BIFI-IQFR (CSIC) and GBs-CSIC, University of Zaragoza, Zaragoza, Spain

**Keywords:** cancer genetics, CHEK2, breast cancer, genetic testing, bioinformatics analysis

## Abstract

Inherited mutations in the *CHEK2* gene have been associated with an increased lifetime risk of developing breast cancer (BC). We aim to identify in the study population the prevalence of mutations in the *CHEK2* gene in diagnosed BC patients, evaluate the phenotypic characteristics of the tumor and family history, and predict the deleteriousness of the variants of uncertain significance (VUS). A genetic study was performed, from May 2016 to April 2020, in 396 patients diagnosed with BC at the University Hospital Lozano Blesa of Zaragoza, Spain. Patients with a genetic variant in the *CHEK2* gene were selected for the study. We performed a descriptive analysis of the clinical variables, a bibliographic review of the variants, and a cosegregation study when possible. Moreover, an in-depth bioinformatics analysis of CHEK2 VUS was carried out. We identified nine genetic variants in the *CHEK2* gene in 10 patients (two pathogenic variants and seven VUS). This supposes a prevalence of 0.75% and 1.77%, respectively. In all cases, there was a family history of BC in first- and/or second-degree relatives. We carried out a cosegregation study in two families, being positive in one of them. The bioinformatics analyses predicted the pathogenicity of six of the VUS. In conclusion, CHEK2 mutations have been associated with an increased risk for BC. This risk is well-established for foundation variants. However, the risk assessment for other variants is unclear. The incorporation of bioinformatics analysis provided supporting evidence of the pathogenicity of VUS.

## Introduction

Breast cancer (BC) remains the second most common cancer worldwide, and it is the leading cause of death by cancer in women (Bray et al., 2018). Inherited mutations account for 5%–10% of BC (in *BRCA1*, *BRCA2,* and other BC susceptibility genes) (Valencia et al., 2017). Pathogenic mutations in the *BRCA1* and *BRCA2* repair genes confer high risks of developing hereditary breast and ovarian cancer (HBOC). A lifetime risk of BC has been well-established for *BRCA1* and *BRCA2* mutation carriers. However, only approximately 25% of cases can be ascribed to *BRCA1* and *BRCA2* mutations (Nielsen et al., 2016). With the advent of next-generation sequencing (NGS) technologies, germline testing for hereditary BC and ovarian cancer (OC) will be extended beyond the analysis of the *BRCA1* and *BRCA2* genes (Desmond et al., 2015). Thus, inherited mutations in other genes of high and moderate penetrance, such as the *CHEK2* gene associated with hereditary BC (Cybulski et al., 2004; Easton, 2004; Weischer et al., 2008), will surely require attention.

*CHEK2* is a tumor suppressor gene that encodes a serine/threonine kinase, Chk2. It is involved in DNA repair, cell regulation, and apoptosis in response to DNA damage (Hirao et al., 2000; Cai et al., 2009; Magni et al., 2014). The heterozygous germline mutation in *CHEK2*, 1100delC, was first identified in 1999 in families with Li–Fraumeni-like syndrome (Bell et al., 1999; Vahteristo et al., 2001). In 2002, it was reported as the cause of BC (Meijers-Heijboer et al., 2002), and subsequent studies have confirmed this association (Easton, 2004; Weischer et al., 2008). Other pathogenic variants have been identified in the *CHEK2* gene, whose prevalence in the general population has been estimated at approximately 1%. The contribution of the *CHEK2* gene as a moderate risk gene for BC has been firmly established by several large-scale sequencing studies that obtain a relative risk of about 2 (Couch et al., 2017; Lu et al., 2019). Some large cases and control studies carried out recently have found rare missense variants in significant excess in *CHEK2*. According to a study which involves 13,087 BC cases and 5,488 controls, the odds ratio (OR) (95% confidence interval, CI) for *CHEK2* rare missense variants was 1.36 (CI: 0.99–1.87) and 1.51 (CI: 1.02–2.24), considering only the functional domains (Han et al., 2013; Decker et al., 2017). Furthermore, in the BEACCON study (“hereditary BrEAst Case CONtrol study”) (Li et al., 2021), which was conducted to investigate the monogenic causes underlying the familial aggregation of BC beyond *BRCA1* and *BRCA2,* the prevalence of rare missense variants in *CHEK2* was 2.11% (122 cases) *versus* 1.24% (71 control cases), with an OR 1.73 (CI: 1.27–2.35). Other studies have shown that *CHEK2* mutation carriers develop ductal carcinomas with positive expression of an estrogen receptor (ER) (Vargas et al., 2011; Gallagher et al., 2020). Thus, the classification of these variants in the *CHEK2* gene poses a challenge, given the conflicting interpretations in hereditary cancer diagnosis (Balmaña et al., 2016; Li et al., 2021).

In this work, a genetic study of a cohort of patients diagnosed and treated of BC (the population is described below in **Materials and Methods**), along with a descriptive analysis of relevant clinical variables, the scrutiny of the variants identified, and a cosegregation evaluation have been carried out to identify the prevalence of missense variants in the *CHEK2* gene. Moreover, a thorough structural/functional analysis is complemented with some bioinformatics and molecular dynamics (MD)-based approaches to help predict the deleteriousness arising from identified mutations in BC patients studied. Protein stability or integrity, interactions with other protein partners, and regulatory and functional sites/domains are key aspects on which mutations can have a direct impact. Available reports on experimental data related to some of these issues, assessed together with insights obtained from sequence conservation analysis, the protein structure itself, and relaxation molecular dynamics (rMD) simulations, enable us to provide here not only a higher confident prediction in most cases but also the rationale behind the verdicts proposed.

## Materials and methods

### Patients

This is an observational, unicentric, retrospective study of a cohort of 396 patients who were diagnosed and treated of BC at the Hospital Clínico Universitario Lozano Blesa of Zaragoza, Spain. A genetic study was performed in all patients from May 2016 to April 2020. The patients were selected due to their personal or family history of cancer, using the criteria for the indication of a germline genetic study recommended by the Spanish Society of Medical Oncology (Llort et al., 2015). Prior to conducting the germline genetic study, all patients received pre-test genetic counseling. The patients were duly informed of the nature and purpose of the genetic study, and all provided informed consent prior to the extraction of the blood sample. After the genetic study, all patients received post-test genetic counseling.

The genetic variants identified were categorized, according to the American College of Medical Genetics and Genomics (ACMG) guidelines, into five categories, namely, pathogenic (class 1), likely pathogenic (class 2), of uncertain significance (class 3), likely benign (class 4), and benign (class 5) (Richards et al., 2015). After such a classification, patients with variants of uncertain significance (VUS) in the *CHEK2* gene were selected for the study. All patients underwent a personal and family medical history analysis. Personal clinical data included the age at the diagnosis of BC, bilaterality, histology and tumor grade, and the subtype and stage, as well as lymph node involvement. A family pedigree of at least three generations was prepared. The patients were asked to provide complete information about cancers in all relatives, including the type of cancer, age at onset, age at death, and the current age of relatives without cancer. A descriptive analysis of the clinical variables, a bibliographic review of the variants identified, a bioinformatics analysis, and a cosegregation study were carried out when possible.

### Genetic study

The exonic and intronic regions with clinical relevance of the following genes were studied by NGS: *BRCA1*, *BRCA2*, *MLH1*, *MSH2*, *MSH6*, *ATM*, *PALB2*, *CHEK2*, *TP53*, *PTEN*, *STK11*, *CDH1*, *NBN*, *BARD1*, *RAD50*, *MRE11A*, *XRCC2*, *BRIP1*, *RAD51D*, *RAD51C*, *CDKN2A*, and *CDK4*. ThermoFisher Ion Torrent equipment was used. The preparation of libraries and their enrichment were done using AmpliSeq technology, and the bioinformatics analysis of the results was performed using Torrent Suite™ (version 5.10.1) and Ion Reporter™ (version 5.10.5.0) software applications.

The panel of genes selected was prepared at the Genetics Laboratory of the Biochemistry Department. Using the technology, 100% coverage of the analyzed genes was achieved, with a 30× depth for 100% of the analyzed sequences. The sequencing performed enabled the study of copy number variations (CNVs) for all the genes analyzed. The identified pathogenic variants were confirmed by Sanger sequencing.

### Descriptive analysis

A descriptive analysis of the collected variables was performed, taking into account the epidemiological, clinical, and those variables related to personal and family history. Nominal qualitative variables are expressed by frequencies and absolute values, and ordinal qualitative variables are represented by percentages of absolute values.

### Cosegregation study

The search for VUS was extended to other relatives affected by cancer if they were available and provided their consent. It was possible to carry out the study in two of the families where this type of variant was identified.

### Bioinformatics analyses on *CHEK2* VUS

Sequence conservation analysis of the Chk2 protein was done after performing a multiple sequence alignment (blastp, BLOSUM62, https://blast.ncbi.nlm.nih.gov/Blast.cgi#,last accessed on 15 November 2021) over the “non-redundant protein sequences (nr)” database (451,821,795 sequences). A maximum of 500 hits (sequences with the highest scores) was setup for the alignment, which led to a minimum identity of 64.8% *versus* the target sequence. The conservation analysis was based on a sequence logo generated (https://webi.berkeley.edu/, last accessed on 15 November 2021) from the resulting multiple alignment.

Pathogenicity prediction verdicts for the analyzed variants were issued through the classification algorithm behind our recently released prediction tool, PirePred (Galano-Frutos et al., 2022), an accurate server that enables predictions for variants of 58 selected genes relevant to newborn screening. The majority vote that the algorithm used relies on 15 renowned prediction tools and optimized cutoff values (Galano-Frutos et al., 2022). PirePred is designed to provide predictions in three different modes, namely, “high-coverage,” “intermediate,” and “low-FPR,” to fit its performance according to the condition (disease) prevalence scenario, i.e., under testing of high-, intermediate-, or low-prevalence populations. Pathogenicity verdicts for the identified VUS are obtained under the modes mentioned.

The variant frequency was evaluated at a worldwide population level by querying the Genome Aggregation Database (gnomAD, https://gnomad.broadinstitute.org/, last accessed on 19 November 2021) and the International Genome Sample Resource (IGSR) from the 1000 Genomes Project database. The data from the latter were accessed through a link to the website of the Ensembl project (https:// www.ensembl.org/index.html, last accessed on 19 November 2021).

Predictions of phosphorylation sites (p-sites) by cognate protein kinases were retrieved from the GPS v5.0 server (Wang et al., 2020) (http://gps.biocuckoo.cn/online.php, last accessed on 22 November 2021) and NetPhos v3.1 (Blom et al., 2004) (https://services.healthtech.dtu.dk/service.php?NetPhos-3.1, last accessed on 22 November 2021).

### Static structural analysis of *CHEK2* VUS

The dimeric Chk2 protein (UniProt code O96017) associated with the *CHEK2* gene is characterized by the presence of an N-terminal serine–glutamine/threonine–glutamine cluster domain (SCD), a middle forkhead-associated (FHA) β-sandwich domain (residues 113–175), and a C-terminal Ser/Thr kinase domain (residues 220–486) (Oliver et al., 2006; Modi and Dunbrack, 2019). Even though no full-length structure experimentally solved is available, partial high-resolution structures of Chk2 encompassing the FHA or kinase domain have been released, e.g., PDB 1GXC (2.7 Å, residues 92–207) (Li et al., 2002) and PDB 2CN5 (2.25 Å, residues 210–504) (Oliver et al., 2006). The static structural analysis performed in relation to the identified Chk2 VUS was based on these two PDB structures. The evaluation of steric (clashes upon amino acid change) and distance/electrostatic features (relative positions to relevant functional spots, cation/π, salt bridge, hydrogen bond interactions, *etc.*), both at the intra and inter-monomeric levels, was accomplished using the molecular visualizer Swiss-PdbViewer v4.1.0 (Swiss Institute of Bioinformatics) (Guex and Peitsch, 1997).

### rMD simulations and trajectory analysis for the assessment of protein stability on *CHEK2* VUS

All-atom 1-µs-length rMD simulations were performed for the wild-type protein and each of the VUS detected in the BC patients. Since protein domains are independent folding units, the FHA domain does not participate at the dimerization interface of Chk2, and the identified VUS are far from the inter-domain interaction region (see the biologic unit in the PDB with the current highest coverage: 3I6U) (Cai et al., 2009); it was decided to simulate the FHA domain independently as a monomer. The starting structure was the one in PDB 1GXC (2.7 Å) (Li et al., 2002), which has a higher resolution than that in PDB 3I6U (3.0 Å). On the other hand, the kinase domain is simulated as a dimer, with the starting structure taken from PDB 2CN5 (2.25 Å) [(Oliver et al., 2006). In this case, the two Mg^2+^ ions (relevant for the catalytic interactions and protein function) and one ADP molecule (substrate) present in each monomer of the protein chains in 2CN5 have been kept in the simulations. Therefore, this ADP molecule has been parametrized. The parametrization procedure and programs used, together with the setup and principal details of the performed rMD simulations, are provided in Supplementary Information Methods.

## Results

Of the 396 patients analyzed in the genetic study, nine genetic variants in the *CHEK2* gene were identified in 10 patients (Table 1). They comprise two pathogenic variants (c.349 A>G, found in two patients, and c-507delT) and seven VUS (c.844C>G**,** c.1033C>T**,** c.300G>T**,** c.1175C>T**,** c.1412C>T**,** c.503C>T, and c.1420C>T). This supposes a prevalence of 0.75% and 1.77%, respectively.

**TABLE 1:** Phenotypic characteristics and family history of probands with either pathogenic/likely pathogenic variants or VUS.

Family no.	Proband						Family history		
	*CHEK2* genotype (NM_007194)	Classification of variants	Age at diagnosis of BC	Tumor phenotype	Bilateral BC	Other tumors	BC in FDR^a^	BC in SDR^b^	Other tumors
1	c.349 A>G	P/LP	62	Triple-negative	No	No	1	0	Renal
2	c.349 A>G	P/LP	64	Luminal B	No	No	1	0	Pancreatic and ovarian
3	c-507delT	P/LP	56	Her2+	No	No	1	1	Ovarian and endometrium
4	c.844C>G	VUS	45	Luminal A	No	No	1	0	Prostate
5	c.1033C>T	VUS	38	Luminal B	No	No	1	0	Pancreatic, melanoma, and endometrium
6	c.300G>T	VUS	54	Luminal B	No	No	1	0	Prostate, pancreatic, and glioblastoma
7	c.1175C>T	VUS	38	Luminal B	No	No	0	2	Gastric and bladder
8	c.1412C>T	VUS	39	Luminal B	No	No	1	1	Prostate and lung
9	c.503C>T	VUS	57	Luminal–Her2+	No	No	2	1	None
10	C.1420C>T	VUS	69	Luminal A	No	No	0	2	Pancreatic and prostate

^a^ BC in FDRs, breast cancer in first-degree relatives.

^b^ BC in SDRs, breast cancer in second-degree relatives.

### Phenotypic characteristics and family history

The phenotypic characteristics of the probands and the family history are summarized in Table 1. The average age of patients with pathogenic or likely pathogenic variants was 60.7 years (from 56 to 64), and none was diagnosed with bilateral BC or had a personal history of other tumors. All patients have a family history of BC in first- and/or second-degree relatives. The family history included one or two of the following types of cancer: pancreatic, ovarian, kidney, and endometrial cancers.

In the case of patients with VUS, the average age was 45.2 years (from 38 to 57), while only one of the probands was a male subject. All patients were diagnosed with a luminal tumor, except one patient who was diagnosed with Luminal–Her2+. In all cases, there was a family history of BC in first- and/or second-degree relatives. The family history included one or more of the following types of cancer: prostate, pancreas, melanoma, lung, gastric, endometrial, cerebral, and urothelial cancers.

### Family description

**FIGURE 1:**
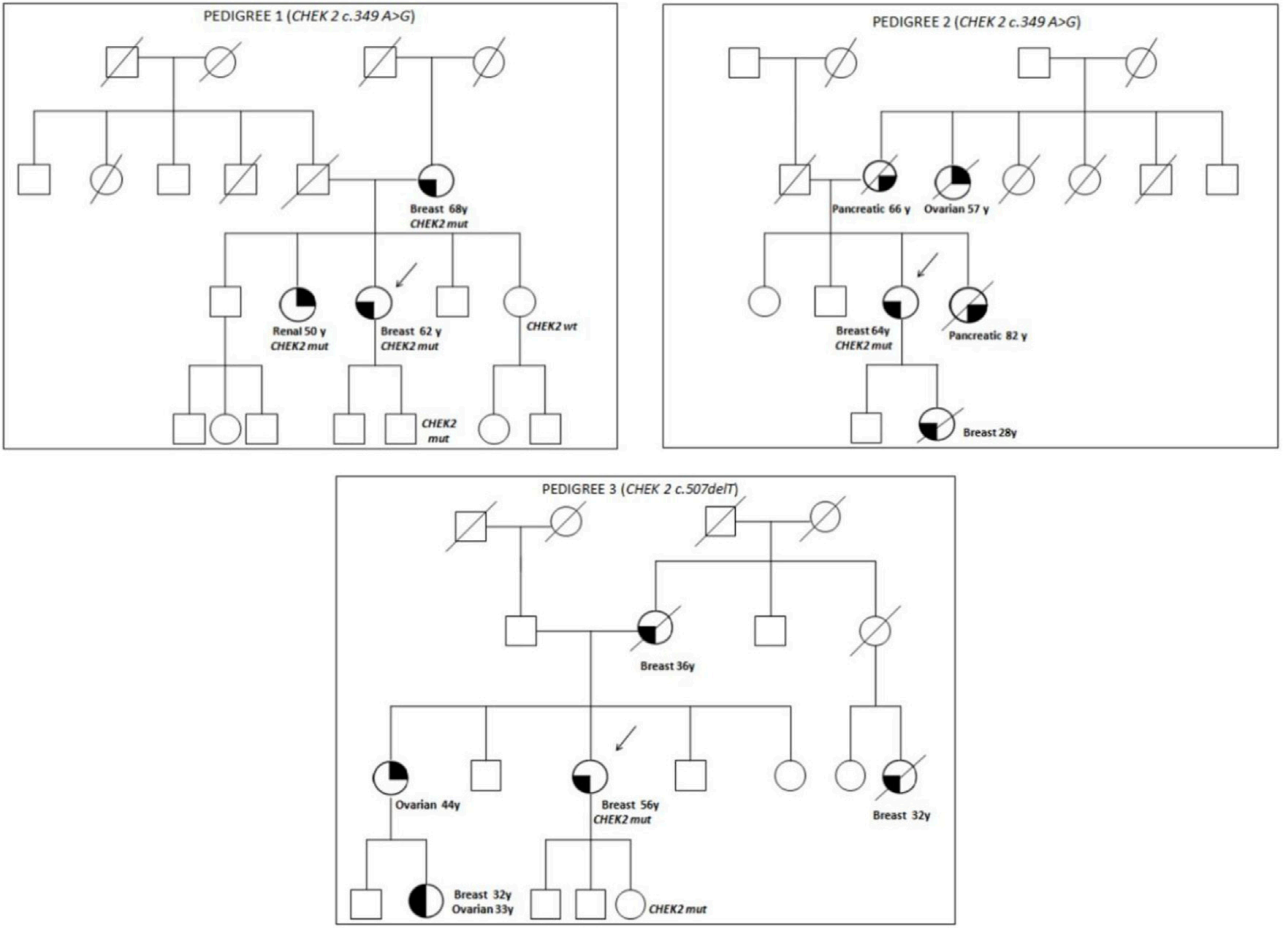
Pedigrees of families 1, 2, and 3.

The family pedigrees of patients with pathogenic variants are shown in Figure 1.

Pedigree 1 (Figure 1): The proband developed triple-negative BC under an age of 62 years. Their mother had BC at the age of 68 years, and their sister developed renal carcinoma approximately at the age of 50 years. Genetic testing revealed the *CHEK2* variant c.349A>G. Through testing the mother, we could define it had been inherited from the proband. In addition, the proband's sister, diagnosed with renal carcinoma, also tested positive for this variant.

Pedigree 2 (Figure 1): The proband developed hormone-responsive BC at the age of 64 years. Their mother and sister developed pancreatic cancer in their 60s and 80s, respectively. A maternal aunt developed OC at the age of 57 years, and the proband's daughter had BC at 28 years. Due to their family history, the proband underwent genetic testing, which led to the detection of the *CHEK2* variant c.349A>G. However, we could not test the affected relatives as they had died. It was not possible either to test the father to check the parental origin of the variant.

Pedigree 3 (Figure 1): The proband had Her2+ BC at the age of 56 years**.** They underwent genetic testing due to a history of breast and ovarian cancers in their family. Their mother was diagnosed with BC at the age of 36 years, and a maternal first-degree cousin, at the age of 32 years; both had died at the time of this study. Moreover, the sister developed OC in their 40s, and the niece (the daughter of her sister) was diagnosed with BC and high-grade serous ovarian carcinoma from the age of 32 and 33 years, respectively. The *CHEK2* variant identified (c.507delT) has been associated with ovarian and endometrial cancers. However, due to different reasons, none of the members affected in this family were studied.

**FIGURE 2:**
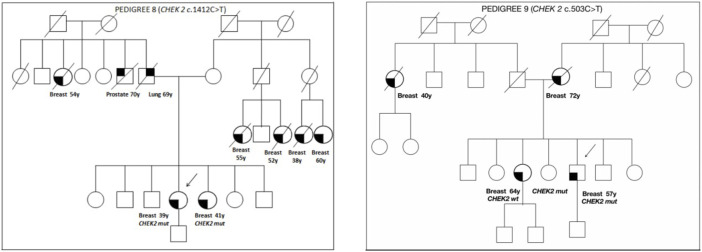
Pedigrees of families 8 and 9.

The family pedigrees of patients with a VUS, in which a cosegregation study was carried out, are shown in Figure 2.

Pedigree 8 (Figure 2): The proband developed hormone-responsive BC under age 39 years. Their sister had BC at a similar age. The *CHEK2* variant c.1412C>T was detected in the proband and then confirmed in their sister. This is a missense variant not present in population databases (it has not been reported in the literature in individuals with *CHEK2*-related diseases). In the maternal side of the family, there were four first-degree cousins who had died because of BC.

Pedigree 9 (Figure 2): The proband is a man who developed BC at the age of 57 years. His mother and sister had BC at the age of 72 and 64 years, respectively. In addition, in the paternal side of the family, there is an aunt who developed BC at the age of 40 years. Genetic testing in the proband revealed the *CHEK2* variant c.503C>T. This variant was subsequently checked in his sister, who was demonstrated not to carry the variant. Such a result enabled us to exclude the variant as a predisposition factor shared by the siblings.

### Sequence conservation analysis

**FIGURE 3:**
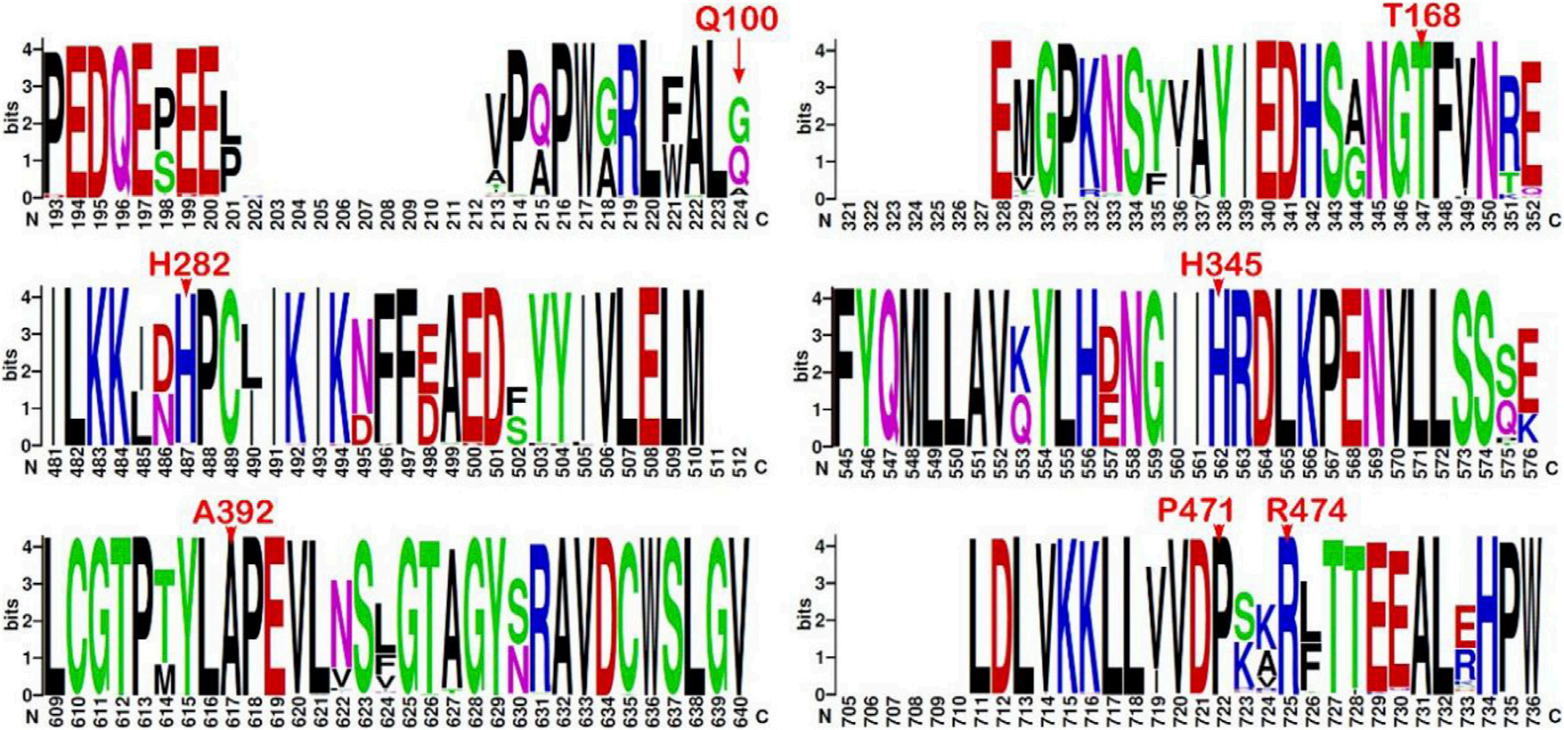
Sequence logo from multiple alignments of *CHEK2* homolog sequences. Sequence conservation represented through a sequence logo obtained from the top 500 (best-scored) hits aligned to the *CHEK2* sequence (minimum sequence identity of 64.8%). Only those fragments containing the mutation sites associated with the VUS identified in the BC patients are depicted (residues indicated in red font above the position). Sequence numbering is not ordered based on the sequence (UniProtKB O96017, isoform 1) but on the alignment.

The multiple sequence alignment of the top 500 best hits against the *CHEK2* sequence (see **Materials and Methods**) indicates that Gln100, associated with the VUS c.300G>T, is the second residue with the highest prevalence at position 100, where Gly predominates and Ala has also been found. Therefore, the conservation at position 100 is moderate. In contrast, all other residues changed in the remaining VUS detected here are highly conserved, namely, Thr168 (c.503C>T), His282 (c.844C>G), His345 (c.1033C>T), Ala392 (c.1175C>T), Pro471 (c.1412C>T), and Arg474 (c.1420C>T) (Figure 3).

### Prediction of pathogenicity using PirePred

PirePred (Galano-Frutos et al., 2022) was originally trained with variants found in a set of genes encoding enzymes related to conditions identified in neonatal screening programs. After its release, its performance was checked on a panel of 10 unrelated genes, and very similar statistics were obtained (not shown). Before using PirePred to evaluate the *CHEK2* variants described here, we tested its performance on a set of *CHEK2* single-nucleotide variants that appear annotated in ClinVar with at least one star, excluding those described as of uncertain significance or having conflicting interpretations of pathogenicity (last accessed on 14 November 2023). All these variants (11 missense and 132 nonsense) were correctly classified by PirePred as either benign (eight missense variants) or pathogenic (three missense plus all nonsense), which indicates that PirePred is an appropriate tool to classify *CHEK2* variants. The Genomic Data Commons Data Portal (https://portal.gdc.cancer.gov/genes/ENSG00000183765) (Grossman et al., 2016) reporting *CHEK2* somatic mutations was also inspected. It reports 126 somatic mutations associated with the *CHEK2* gene (100 missense, 17 frameshift, and 9 nonsense), but none of those missense mutations occur at the same positions, as those of the mutations investigated in this study.

The prediction verdicts issued by the PirePred classification algorithm for *CHEK2* VUS identified in the BC patients of this genetic study are summarized in Table 2 for the three PirePred prediction modes defined in **Materials and Methods**. This table also provides the original verdicts issued by the 15 predictive tools used to train the classifier. Most variants (Arg474cys, Ala392Val, His345Tyr, His282Asp, and Thr168Ile) are classified by PirePred as pathogenic under any of its three prediction modes, namely, high-coverage, intermediate, and low FPR. This indicates that the variants are considered to be pathogenic, with the server operating at an FPR of 11.5%. On the other hand, the variant Pro471Leu is classified as pathogenic under the high-coverage and intermediate modes but as VUS at the low-FPR mode. This indicates that the variant is classified as pathogenic with the server operating at an FPR of 20%. In contrast, Gln100His is classified as VUS by any of the three operating modes.

**TABLE 2:** PirePred pathogenicity classification of VUS and verdicts of the individual predictors combined^a^.

Variant	PirePred			Individual predictors														
	P-to-VUS threshold^b^	B/(B+P) rate^c^	B/VUS/P^c^	SIFT	PolyPhen2	LRT	MutationTaster	MutationAssessor	PROVEAN	Meta SVM	M-CAP	Revel	MutPred	MVP	DEOGEN2	ClinPred	LIST-S2	CADD
	0.4		VUS															
Q100H	0.24	0.47	VUS	**0.75^d^**	**0.6^d^**	**0.3^d^**	0.81	0.54	**0.56^d^**	0.84	0.59	0.71	**0.52^d^**	0.93	**0.53^d^**	0.38	0.71	**0.6^d^**
	0.08		VUS															
	0.4		P															
T168I	0.24	0.00	P	0.78	0.92	0.84	0.81	0.9	0.86	0.98	0.89	0.99	0.87	0.98	0.94	0.85	0.74	0.71
	0.08		P															
	0.4		P															
H282D	0.24	0.07	P	0.78	0.75	0.84	0.81	0.85	0.98	0.87	0.75	0.96	0.96	0.98	**0.78^d^**	0.95	0.73	0.85
	0.08		P															
	0.4		P															
H345Y	0.24	0.07	P	0.91	0.97	0.84	0.81	0.86	0.87	0.91	0.83	0.98	0.99	0.97	**0.74^d^**	0.89	0.98	0.84
	0.08		P															
	0.4		P															
A392V	0.24	0.07	P	0.91	0.78	0.84	0.81	0.95	0.72	0.93	0.88	0.95	NA^e^	**0.72^d^**	0.82	0.75	0.96	0.83
	0.08		P															
	0.4		P															
P471L	0.24	0.13	P	0.78	0.84	0.84	0.81	0.9	0.97	0.9	0.78	0.82	0.89	**0.73^d^**	**0.75^d^**	0.88	0.91	0.83
	0.08		VUS															
	0.4		P															
R474C	0.24	0.00	P	0.91	0.97	0.84	0.81	1	0.95	0.96	0.82	0.97	1	0.95	0.83	0.98	0.94	0.8
	0.08		P															

^a^ Predictors included in the PirePred classification algorithm (Galano-Frutos et al., 2022) selected from the dbNFSP v4.1a (Liu et al., 2011) repository.

^b^ P-to-VUS, thresholds established in PirePred (Galano-Frutos et al., 2022) for the three classification modes setup, namely, high-coverage (0.4), intermediate (0.24), and low-FPR (0.08).

^c^ Calculated rate used to compare with the threshold values established in PirePred. B, P, and VUS stand for benign, pathogenic, and variant of uncertain significance verdicts, respectively, obtained from the selected predictors.

^d^ Benign verdicts issued by the selected predictors are highlighted in bold.

### Allele frequency in genomic databases

We searched through worldwide population samples in genomic databases to find out whether the identified variants had been previously detected and what their prevalence was. In the database of the 1000 Genomes Project, which is—at the moment—10 times smaller than the gnomAD version searched “v2.1.1 (controls)” (6,020 vs. 60,146 samples, respectively), only the variant Arg474Cys has been reported (in canonical transcript ENST00000404276.6, https://www.ensembl.org/Homo_sapiens/Transcript/Haplotypes?g=ENSG00000183765;r=22:28687743-28741820;t=ENST00000404276), with only one carrier (European) found (an allele, with a frequency of 1.66 × 10^−4^ in the whole population). On the other hand, two of the variants, Ala392Val (seven alleles, six European and one East Asian, https://gnomad.broadinstitute.org/variant/22-29091782-G-A?dataset=gnomad_r2_1_controls) and Arg474Cys (one allele, European, https://gnomad.broadinstitute.org/variant/22-29090061-G-A?dataset=gnomad_r2_1_controls), appear to be reported in gnomAD, whose allele frequencies amount to 6.4 × 10^−5^ and 1.02 × 10^−5^, respectively (in the same transcript).

### Reported phosphorylation sites and prediction

Some phosphorylation sites important for homodimerization and activation are present in Chk2 (Matsuoka et al., 2000; Lee and Chung, 2001; Tosti et al., 2004). To assess whether the only modifiable amino acid (Thr168)—among those changed in the mutations identified in BC patients—has been reported as PTM-susceptible and functionally relevant, an exhaustive revision of the bibliography and PTM databases was done. The databases revised included iPTMnet (https://research.bioinformatics.udel.edu/iptmnet/), GlyGen (https://glygen.org/home/), MetOSite (https://metosite.uma.es/), and PhosphoSite (https://www.phosphosite.org/) (all last accessed on 22 November 2021). None of them report on the phosphorylation of this residue or any other PTM at this position. Moreover, both the online servers GPS v5.0 (Wang et al., 2020) and NetPhos v3.1 (Blom et al., 2004) predict no phosphorylation on Thr168.

### Static structural analysis of *CHEK2* VUS

One approach to attempt to extract clues about the impact of an amino acid substitution on the protein function may be the analysis of conserved motifs and their proximity (spatial) to structural elements associated with, for instance, the catalytic center, dimerization, partner interaction interfaces, or other similarly relevant features. Likewise, preliminary stability analysis can be done based on criteria such as disruption of native interactions (e.g., hydrogen bonds, salt bridges, and cation/π) or *de novo* formation of some of them, the formation of cavities, or the likelihood of significant steric clashes, just to mention a few.

**FIGURE 4:**
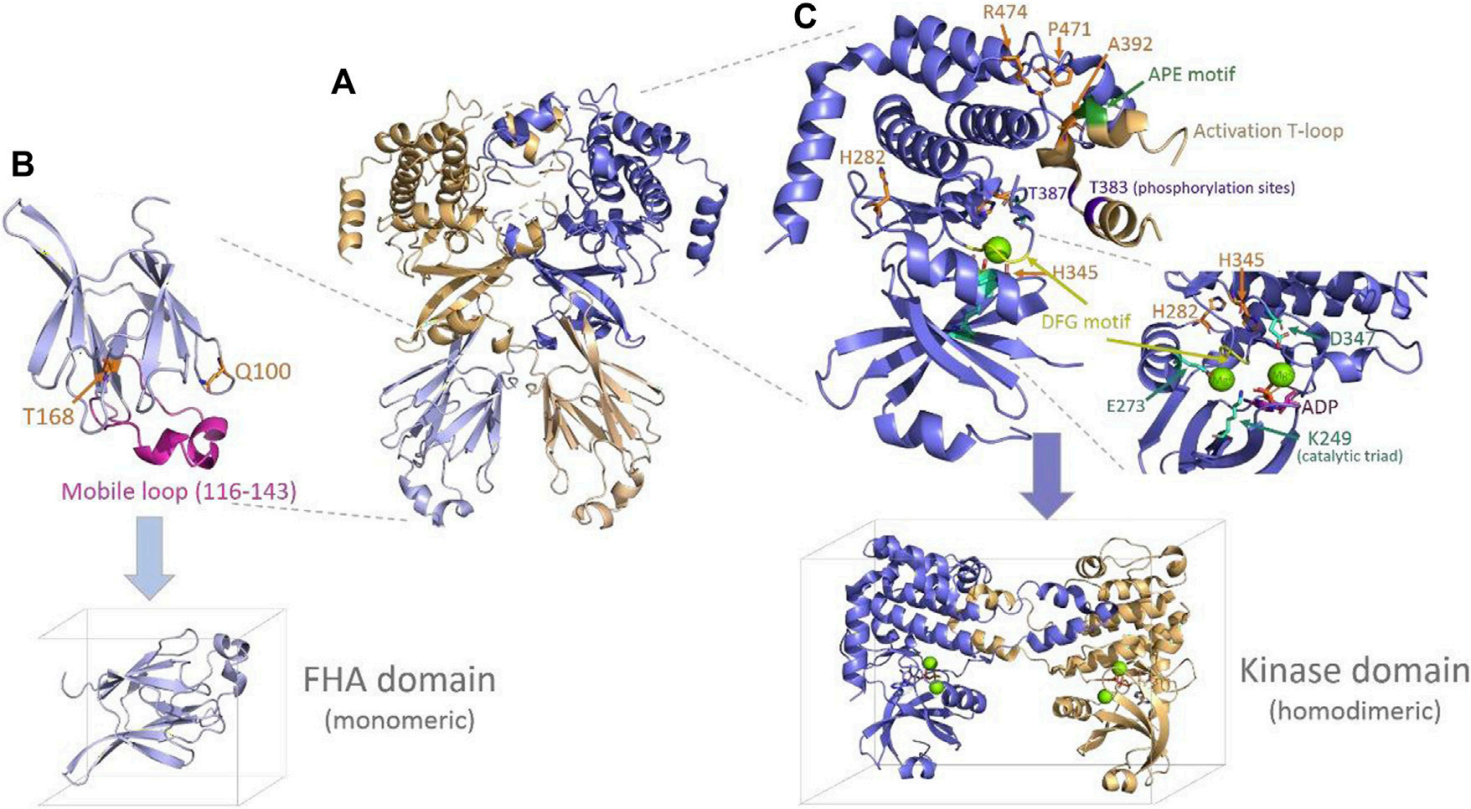
Chk2 structure, domains, and main features. **(A)** Chk2 structure (cartoon) with the highest coverage (homodimeric biological unit) released in PDB 3I6U (Grossman et al., 2016). **(B)** FHA domain (PDB 1GCX (Li et al., 2002), cartoon) with the mutation spots of the VUS highlighted in orange (sticks) and the flexible long loop highlighted in magenta. The simulation box below indicates the way in which this domain has been simulated (monomeric). **(C)** Kinase domain representation (cartoon) including one monomer and the activation of the T-loop from the second monomer, as solved in PDB 2CN5 (Oliver et al., 2006). The mutation spots of the VUS are highlighted (sticks in orange), as well as the main conserved motifs and residues in the catalytic site and the T-loop. One molecule of substrate ADP (sticks in magenta) and two magnesium ions (green spheres) located at the catalytic site—as per 2CN5—are also depicted. The simulation box below indicates the way in which this domain has been simulated (homodimeric, with Mg^2+^ ions and the ADP substrate).

The Chk2 protein presents some conserved motifs and determinant features for homodimerization, activation, and functioning (Oliver et al., 2006). Upstream of the FHA domain appears the conserved Thr68 residue that is phosphorylated by ATM/ATR kinases to promote dimerization and trans-activating phosphorylation of other two conserved residues, Thr383 and Thr387. These latter residues are placed in a long loop, so-called “activation T-loop,” which starts by a conserved triad (DFG motif: Asp368–Phe369–Gly-370) that helps conform to the catalytic site (Figure 4). The T-loop also includes a conserved triad downstream (APE motif: Ala392–Pro393–Glu394), likely crucial for the dimerization interaction. Other conserved spots include the catalytic residues, Lys249, Glu273, and Asp347, and charged residues that appear coordinated to magnesium (Mg^2+^) in PDB 2CN5 (Oliver et al., 2006) and seem to be crucial for the catalytic function (phosphorylation) of the protein (see Figure 4). Here, inspection of the Chk2 structure (PDBs 3I6U, 1GXC, and 2CN5) and some local analyses were done at the mutation sites to provide—when it is the case—structural insights into the relationship with the abovementioned crucial features in Chk2.

Starting with mutations located in the FHA domain, Gln100 is placed at the beginning of a small loop connecting strand 1 and strand 2. Its side chain is exposed to a solvent, forms a hydrogen bond (H-bond) with Asn196, and is far (>10 Å) from any atom of the opposite FHA domain. Its replacement by histidine at this position would imply the introduction of a positive charge without an apparent destabilizing effect on the protein. Substitution of the H-bond with Asn196 could take place through one of the nitrogen atoms of the imidazole ring. In the case of Thr168, it appears to be located in a long loop connecting strand 5 and strand 6. The side chain is buried, forming an apparently stabilizing local H-bond network with Asp162, Ser164, and His143, and is far from any other domain or inter-monomeric surface. Its substitution with an isoleucine residue represents the introduction of a bulkier residue and, at the same time, the disruption of the referred stabilizing H-bond network. None of the two residues described, Gln100 and Thr168, seem related—at least in an obvious manner—to any of the previously mentioned conserved motifs and functional features in the Chk2 protein.

Of the mutations located in the kinase domain, His282 is at the protein surface, putatively forming a cation/π interaction with Tyr337. Substitution of His282 by aspartate would imply the disappearance of the positive charge of the histidine and the introduction of a smaller, negatively charged residue, which may affect the abovementioned interaction with Tyr337. In addition, there appear to be no neighboring residues suitable for hydrogen bonding. His282 does not appear to be directly related to any of the conserved motifs and key functional elements either. A second mutation at the kinase domain affects His345, a residue which is part of the conserved motif HRD (His345–Arg-346–Asp347) in the catalytic loop. In this motif, the key catalytic residue Asp347 (Oliver et al., 2006; Modi and Dunbrack, 2019) establishes an H-bond with His345. In addition, His345 forms a fair H-bond (∼2.7 Å) with the backbone of Asp368, located in the activation T-loop crucial for homodimerization. The replacement of His345 by a bulkier tyrosine residue in a buried spot may be difficult to accommodate properly. A third mutation in the kinase domain involves Ala392, which is part of the conserved APE motif (Ala392–Pro393–Glu394) also located in the long T-loop. The substitution of alanine introduces a bulkier valine residue at a spot where Ala392 appears to be in close contact with some residues of the partner monomer (dimerization interface), particularly with Arg474, which is one of the mutated residues (Arg474Cys) detected among the BC patients analyzed here. A fourth mutation refers to Pro471, a partly exposed residue, the buried face of which is also packed with Arg474 (at the same monomer). Both Pro471 and Arg474 integrate an 8-residue turn (Val468 to Phe475) connecting short α-helices. The turn gets packed against the T-loop (from the other monomer), which appears in the structure as giving a hug to its partner monomer, thus favoring homodimerization. Replacing Pro471 by a leucine residue does not bring about a significant steric issue, but it may lead to an increased flexibility in the turn, which could impair the inter-monomeric packing. Finally, a fifth mutation takes place in Arg474, which, in addition to what has already been indicated above, forms a cation/π interaction with Trp411 (at the same monomer) and a salt bridge with Glu394 (at the partner monomer). Replacement of Arg474 with a cysteine residue will remove all the chances to form the indicated stabilizing interactions of Arg474.

### rMD simulations and stability analysis

MD-based approaches have become reliable tools increasingly used to complement experimental studies at different spatiotemporal resolutions. They have also opened the possibility to qualitatively and quantitatively investigate systems or phenomena not amenable to experiments. The utility of rMD-based approaches to study mutation effects on phenotypes has been summarized elsewhere (Galano-Frutos et al., 2021) and previously applied to the interpretation of variants related to hypertrophic cardiomyopathy (Suay-Corredera et al., 2001). Using this method, studies have focused on issues such as protein stability or partner interaction, helping to decipher the underlying mechanisms through which mutations impair the protein function.

Here, a statistical analysis of stability on the VUS detected in BC patients is addressed through 1-µs-length rMD simulations at three different temperatures (see **Materials and Methods** and Supplementary Table S1). A global destabilization (unfolding) has not been observed over this simulation time for any of the two domains, except for wild-type FHA at 438K (the β-sandwich core unfolds mostly or completely; see Supplementary Figure S1 and Supplementary Figure S2), despite the fact that high temperatures were used to speed up the unfolding kinetics (398, 418, and 438 K for the FHA domain, and 358, 378, and 398 K for the kinase domain). Nevertheless, the simulated time did suffice to observe the local destabilizing effects introduced by the mutation for most of the simulated VUS, as shown below. Therefore, the main results presented here focus on the stability analysis around the mutation site, even though many structural properties have also been calculated on the entire domains in order to have a more comprehensive view of the impact brought about by the mutations.

#### WT domains

Since WT variants should remain stable or at least more stable than most mutants, the WT domain trajectories obtained (three replicas at each temperature) constitute the references with which the mutant trajectories are compared in the subsequent analyses. In the case of the FHA domain, at 398 and 418 K, some global and local properties compiled over time around the mutation sites showed some potentially misleading results (see Supplementary Figures S3–S4a1-a5, c1-c5, e1-e3). On one hand, some global plots may indicate that this domain retains its main folding shape (TM score >0.5), the secondary structure (coil and α+β structure; see Supplementary Figure S1), and the initial network of H-bonds (intra-protein and with water) for each of the three replicas simulated (Supplementary Figures S3, S4a3, c2-c5). However, properties such as RMSD, the fraction of native contacts, SASA, and the radius of gyration (Supplementary Figures S3, S4a1, a4, a5, c1) show certain variations along time, which could be considered triggering some unfolding, but it is not the case (see final frames of trajectories given in Supplementary Figure S1). Such a variability is owed here to the presence of a long flexible loop (Gly116 to His143) connecting strands 3 and 4 (see Figure 4), which is highly mobile (see RMSF plots, Supplementary Figures S3, S4a2). Instead, at 438 K, as mentioned above, the WT FHA domain becomes destabilized, and the β-sandwich core is mostly or completely lost (Supplementary Figure S1). In the kinase domain, even though the activation T-loop is a long one (Figure 4), the situation is different. In such case, the domain is simulated as a dimer, and the T-loop does not appear loose but partly packed onto the second monomer, as described above. At any of the three temperatures simulated for the kinase domain (358, 378, and 398 K; Supplementary Table S1), the WT variant does not show global unfolding or impairment of the dimerization interface (separation of the monomers, see last frames of trajectories in Supplementary Figure S2). Details about the global and local behavior of the WT kinase domain are provided below throughout the comparative analyses performed on the VUS simulated for this domain.

#### Gln100His (FHA domain)

At 398 K and at 418 K, Gln100His shows similar global stability (Supplementary Figures S3 b1-b5, d1-d5, f1-f3 and Supplementary Figures S4 b1-b5, d1-d5, f1-f3, respectively) as the WT domain at the corresponding temperature (see above), with the high flexibility of the long loop still being present (see RMSF plots). However, there are some *a priori* local differences (not significant), in particular, in the fraction of native contacts when compared to WT (plots in **f1–f3** *versus* **e1–e3** in Supplementary Figures S3, S4, respectively), which are related to the substitution of one glutamine by a slightly shorter histidine. Comparative plots obtained from the 2D-RMSD-based local clustering for this variant are given in Supplementary Figure S5. The plots at 398 K and 418 K (Supplementary Figures S5 a1-a3, b1-c3) do not show any local instability along the whole trajectories (only the basal cluster or a few point departures—which get immediately back to it—are observed) for any of the three replicas simulated in both domains (WT and mutant, stable in 3/3 cases, 100%). The β-sandwich core of the FHA domain remained intact in all the cases (Supplementary Figure S1) so that the only significant variability associates with the mobile long loop (Figure 4). At 438 K, the scenario is different because, as described above, the WT domain turned out unstable (sustained departure from the basal cluster, Supplementary Figure S5 c1-c3), while the mutant surprisingly remained stable in 100% of the cases (3/3 replicas).

#### Thr168Ile (FHA domain)

Supplementary Figures S6, S7 include some global and local properties calculated along the trajectories for this variant *versus* WT. Globally, at 398 (Supplementary Figure S6) and 418 K (Supplementary Figure S7), the mutated domain remains folded (see RMSDs <1.0 nm, TM scores >0.5, fractions of native contacts >0.6, and constant intra-protein and protein–water H-bonds), with higher variability being around the already referred long loop (see RMSF and Rg plots). Some individual replicas, e.g., replicas 1 and 3 at 398 K, and replicas 2 and 3 at 418 K, show higher variability in this loop, associated with the transient formation of short α-helical elements on it. Locally, the substitution of Thr168 by an isoleucine causes an *a priori* loss of H-bonds and native contacts compared to WT, which is reflected in plots **f1–f3** *versus* **e1–e3** of Supplementary Figures S5, S6, respectively. However, the lower number of native contacts established by Ile168 remain nearly constant along time, except for replica 1 at 398 K (Supplementary Figures S5 f3) and replica 3 at 418 K (Supplementary Figure S6 f3), where a significant decrease is observed at the end of the trajectories. At the high temperature of 438 K, the mutant remained folded, as shown in Supplementary Figure S1 (the β-sandwich core is still well-folded in all replicas), but since the WT domain did not, a comparative figure like those obtained for the lower temperatures has not been obtained. The 2D-RMSD-based local clustering plots obtained for this variant *versus* WT indicate that at 398 K (Supplementary Figures S8 a1-a3), only replica 1 shows a clear destabilization (Supplementary Figure S8 a1). In this case, replica 1, both from the WT and mutant domains, consistently moved away from the basal conformation (cluster 1). In the other two comparative plots (Supplementary Figure S8 a2, a3 **)**, it is observed that both the WT and mutant domains remained in the basal cluster or in one very close to it. Thus, it seems that at 398 K, both proteins remain mostly stable (2/3, 67%). The increase in temperature up to 418 K (Supplementary Figure S8 b1-b3) also did not lead to sustained destabilization, even if a higher frequency of conformational exchange is observed between the basal cluster and some others very close to it. Here, replica 3 of the mutant (Supplementary Figure S8 b3) was the only one that moved away from the initial clusters, showing sustained destabilization at the end of the trajectory. Thus, at 418 K, in 2/3 of the replicas (67%), the mutant remains locally stable. At 438 K, surprisingly, the mutant also shows local stability in 67% of the cases since only replica 3 shows consistent separation from cluster 1.

#### His282Asp (kinase domain)

The variant H282D shows profiles of high conformational stability, both globally (see RMSDs in Supplementary Figure S9 a1-c1) and locally (see 2D-RMSD-based clustering in Supplementary Figure S10). As for the WT domain, the variability captured (RMSD <1.0 nm for all replicas, Supplementary Figure S9 a1-c1) did not lead to significant impairment of the ternary structure of any of the chains nor the dimerization interface (in 3/3 replicas, the dimer remained associated at any temperature, see Supplementary Figure S2). Moreover, plots obtained from the local clustering analysis showed, in no case, deviations from the basal cluster (cluster 1, Supplementary Figure S10), revealing that no significant instability occurs within the radius of 1 nm around the Asp282 residue. Thus, the amino acid change of an aspartic residue by a histidine at position 282 was tolerated in 100% of the simulated replicas.

#### His345Tyr (kinase domain)

Unlike the previous variant, H345Y shows a less regular stability. At 358 K, the variant exhibits a high-stability profile both globally (RMSD <1.0 nm for all replicas, Supplementary Figure S9 a2) and locally (only the basal cluster is visited as per the 2D-RMSD-based clustering plots in Supplementary Figure S10). At this temperature, two of the three replicas' run ended up with the dimer in its “bound” state (B in 2/3), while, in one replica, separation between the chains took place (“unbound,” see Supplementary Figure S2). At 378 K, a higher global variability is captured in the RMSD plots given in Supplementary Figure S9 a2 (replicas r1 and r2 overcome at some point the RMSD of 1 nm), which is owed mainly to the local rearrangements close to the mutated position. Chain B in replica 1 and chain A in replicas 2 and 3 exhibited sustained separation from cluster 1 in the local clustering plots (Supplementary Figure S11 b1, b2). As at 358 K, the dimerization interface was not affected in two of the three replica run (B 2/3, Supplementary Figure S2). Intriguingly, at a higher temperature of 398 K, H345Y does not show significant higher local instability than the WT domain, as expected based on the results at 378 K. Even though separation from cluster 1 occurs in chains A and B of replica 2 and in chain B of replica 3 (Supplementary Figure S11 c2, c3), the variant remains in these cases in a conformation (cluster 2) very close to the native basal cluster. Replica 1, instead, showed no local instability around the incorporated Tyr345 (Supplementary Figure S11 c2) but exhibited a higher global RMSD associated with variability in other regions, in particular loops (not shown). However, at this temperature the dimer dissociated in all the replicas (U in 3/3), as shown in the conformation of the last frame of the trajectories (Supplementary Figure S2).

#### Ala392Val (kinase domain)

As described above, mutation A392V is the only mutation placed in the activation T-loop, a key element for dimerization of the Chk2 protein. Substitution of alanine by a bulkier valine only in the loop section, where it packs with residues of the other monomer, makes dimerization a matter of greater concern than in the variants described above. Indeed, only in one replica (r3 at 358 K)—regardless of the temperature—did the dimeric structure remain associated until the end of the rMD trajectory (U in 2/3 of replicas at 358 K, and in 3/3 of replicas at 378 and 398 K, see Supplementary Figure S2) so that the mutation seems to affect the dimerization interface. Globally, the RMSD plots obtained for this variant (Supplementary Figure S9 a3-c3) indicate that—at any temperature—two of the three replicas run show higher variability than the WT domain (RMSD >1.0 nm) along the major part of the trajectories. Locally, the 2D-RMSD-based clustering plots (Supplementary Figure S12) indicate that the variant is already unstable at 358 K in at least two of the three replicas simulated (2/3 unstable, 67%). At 358 K, considering the mutation effect on at least one of the monomeric chains, only replica 3 remained locally stable (Supplementary Figure S12 a3), while at 378 K, replica 2 seemed to be less affected. At the higher temperature, all the replicas of the mutant experienced destabilization (separation from the basal cluster), although, here, the effect is associated with the temperature because the WT domain also appears destabilized in the three replicas.

#### Pro471Leu (kinase domain)

As described above, P471L brings about an amino acid change at a spot appearing within the interacting distance to Ala392 and Arg474 residues. The dimerization interaction, as shown for A392V (and as shown for R474C), is, therefore, also a crucial element to be taken into account in the analysis of this variant. In this case, the dimeric kinase domain remains associated only in one of the simulated replica run at 358 and at 378 K (dissociated in 2/3 replicas), while at 398 K, intriguingly, the dimeric structure was conserved in two replicas (Supplementary Figure S2). Global RMSD plots given in Supplementary Figure S9 a4-c4, indicate that, only in a few cases, did replicas of the mutated domain present a higher distortion from the crystal structure of the WT domain, namely, r3 at 358 K and r1 and r2 at 398 K. The local analysis (2D-RMSD-based clustering plots, Supplementary Figure S13), however,reveals that at 358 K, the mutant showed instability (sustained separation from cluster 1) in all the replicas (3/3 unstable, 100%), whereas the WT domain remained stable in all of them (Supplementary Figure S13 a1-a3). At 378 and 398 K, both the mutant and the WT domain became unstable in all the replicas (Supplementary Figure S13 b1-b3 and Supplementary Figure S13 c1-c3).

#### Arg474Cys (kinase domain)

The R474C variant was also mostly deleterious for dimerization in the kinase domain (dissociated in one, three, or two of the replicas run at 358, 378, and 398 K, respectively, Supplementary Figure S2). Likewise, the local clustering plots given in Supplementary Figure S13 show that the destabilizing effect of mutation is significant in all the replicas at all the temperatures; the WT domain, in contrast, remained stable around Arg474. Globally, only in one replica (replica 1 at 378 K) did the conformational distortion from the crystal structure overcome the threshold of RMSD >1.0 nm so that the mutation effect in this variant seems to be more relevant locally and also for dimerization.

## Discussion

The risk of developing BC has been well-established in patients with founder mutations. Most of the data reported on *CHEK2*-associated BC are largely based on studies of protein-truncating variants, in particular the *CHEK2* c.1100delC variant, which is found fairly frequently in Northern European populations (Yang et al., 2012). Otherwise, some multi-gene panel testing studies have identified missense variants which may be associated with a risk of BC (Couch et al., 2017). However, in the majority of missense variants, the risk results are unknown, which is why such variants are referred to as VUS (Easton et al., 2015). However, a recent large case–control study of the Breast Cancer Consortium has established the evidence of an increased risk of BC in rare missense variants in *CHEK2* (Gallagher et al., 2020). In addition, as it occurs in our study, these variants have a stronger association with ER-positive BC than with ER-negative BC (Gallagher et al., 2020). This fact is confirmed in the BEACCON study (Li et al., 2021).

We do not have evidence of the optimal clinical follow-up in carriers of mutations in moderate penetrance genes. In this context, the main clinical guidelines recommend annual screening for women with an estimated cumulative vital risk of ≥20–30%, according to family history, starting at 30–40 years. The cumulative lifetime risk for carriers of mutation in *ATM*, *CHEK2*, and *PALB2* genes approaches or exceeds 30% and justifies the recommendation of the early use of mammography and magnetic resonance imaging (MRI), starting at the age of 30 years in patients with *PALB2* mutations and at the age of 40 years in those carrying *ATM* or *CHEK2* mutations (Tung et al., 2016).

Regarding hormone receptor and Her2 status, according to the data on a study of the German Consortium for Hereditary Breast and Ovarian Cancer, a high *CHEK2* mutation prevalence was observed in patients with Her2-positive tumors compared with patients with Her2-negative tumors (5.2% vs. 2.3%; *p*-value <0.001). In addition, the *CHEK2* gene showed significantly higher mutation rates in patients with no triple-negative BC *versus* patients with a triple-negative BC tumor phenotype (3.3% vs. 0.8%, *p*-value = 0.002). This same study detected the association of *CHEK2* with bilateral BC (Hauke et al., 2018). In our series, we could not confirm these associations because of the small sample size, although one of the patients with a pathogenic variant developed triple-negative BC and another patient developed Her2-positive BC.

Furthermore, other studies have determined the importance of the genetic background to risk modification in hereditary cancer. They have found that the risks associated with germline mutations and the cancer family history act together (Byrnes et al., 2008; Gronwald et al., 2009). In the current study, we found phenotypic heterogeneity among patients carrying the same *CHEK2* mutation, which has potentially important clinical implications when evaluating the risk of developing cancer.

Likewise, we report the prevalence of *CHEK2* variants found in our population, which, in the case of pathogenic mutations, amounts to 0.5%. The pathogenic missense variant c.349A>G was found in two families. This variant was previously associated with BC (Southey et al., 2016). There are also data in the literature that confirm its association with an increased risk of developing prostate cancer (Brandão et al., 2020). However, in our study, this variant was found in a patient with renal carcinoma and in a proband with a strong family history of pancreatic and ovarian cancers (see Table 1 and Figure 1; pedigree 1 and pedigree 2). However, there are no data in the literature on the risk of developing these types of cancer in patients with this specific mutation.

The other *CHEK2* pathogenic variant detected was c.507delT, a frameshift mutation which was found in a family with a history of BC and OC (Figure 1, Pedigree 3). The *CHEK2* variant c.507delT was reported in a family with BC, and it is possibly related with other tumors (Manoukian et al., 2011). In the family analyzed, we could not test other relatives affected.

In relation with the risk of developing cancers other than BC, such as colon or prostate cancer, most of the studies have relied on two common alterations, c.1100delC and c.470T>C (Cybulski et al., 2004; Suchy et al., 2010). In this case, the risks conferred by other variants identified could not be assessed. Moreover, *CHEK2* variants have been observed in individuals with endometrial and ovarian cancers, but the associated risk due to *CHEK2* mutations is currently unclear (Einarsdóttir et al., 2007; Pennington et al., 2013).

For VUS, cosegregation analysis in selected families may help understand whether a variant plays a role in developing cancer. Cosegregation is, however, just one additional tool, which should never provide a definite conclusion on the pathogenicity or neutrality of a variant (Zuntini et al., 2018). Furthermore, this type of studies is often difficult to carry out due to the geographic dispersion of families, lack of collaboration, or death of relatives. Here, a cosegregation study has been carried out in two families, one of which showed the identified variant cosegregated with BC. No additional follow-up strategies have been established for our patients with the *CHEK2* VUS.

On the other hand, information available both in ClinVar (https://www.ncbi.nlm.nih.gov/clinvar/) (Landrum et al., 2020) and VarSome (http://varsome.com.) (Kopanos et al., 2019) is scarce and mostly leads to classify most of the variants analyzed here as uncertain (see Table 3). Thus, regarding this, we carried out a battery of bioinformatics and modeling analyses in order to obtain computational supporting evidence (as defined by the ACMG) (Richards et al., 2015) on either the pathogenic or benign characteristic of the variants found in the cohort of 396 patients diagnosed and analyzed here. The results of the analyses are summarized in Table 3.

**TABLE 3:** Summary of bioinformatics predictions on VUS found in *CHECK2*.

Bioinformatics analysis									
Variant	Replacement (protein domain)	Varsome^a^	ClinVar	Sequence conservation	% in GnomAD (in 1000 Genome project)^b^	Phosphorylation	PirePred classification	Structural insight	rMD
c.300G>T	Gln100His (FHA)	Uncertain significance	Not reported	Moderate	NA (NA)	NA	VUS	Q100 is completely solvent exposed	Does not induce unstability
								Unlikely to affect stability, dimerization or catalysis	
c.503C>T	Thr168Ile (FHA)	Uncertain significance/P (for one user: LP)	Uncertain significance	High	NA (NA)	NOT	Pathogenic^c^	T168 is buried and forms a H-bond with conserved H143. Variant may affect local stability	Induces less severe unstability
c.844C>G	His282Asp (kinase)	Likely pathogenic (predicted splicing)	Not reported	High	NA (NA)	NA	Pathogenic^c^	H282 is solvent exposed, forms cation/π interaction with conserved residue Y337. Not obvious how the variant can affect stability, dimerization or catalysis	Does not induce unstability
c.1033C>T	His345Tyr (kinase)	Uncertain Significance/LP	Uncertain significance	High	NA (NA)	NA	Pathogenic^c^	H345 forms H-bond with conserved catalytic residue D347. Variant may affect catalytic process	Induces unstability
c.1175C>T	Ala392Val (kinase)	Uncertain Significance/LP	Conflicting interpretation of pathogenicity	High	<0.0001 (NA)	NA	Pathogenic^c^	A392 is packed with P471, and R474 from the other monomer. Variant may affect dimerization	Induces unstability
c.1412C>T	Pro471Lys (kinase)	Uncertain Significance/LP	Conflicting interpretations of pathogenicity	High	NA (NA)	NA	Pathogenic^d^	P471 is packed with R474 in the same monomer. Variant may affect dimerization and local stability	Induces unstability
c.1420C>T	Arg474Cys (kinase)	Uncertain Significance/LP	Conflicting interpretations of pathogenicity	High	<0.0001 (<0.0002)	NA	Pathogenic^c^	R474 is buried, forms cation/π with W411 and salt bridge with conserved E394 from other monomer. Variant may affect dimerization and local stability	Induces unstability

^a^ Somatic.

^b^ GnomAD: The Genome Aggregation Database.

^c^ Pathogenicity prediction issued with PirePred operating at aFPR of 11.5%.

^d^ Pathogenicity prediction issued with PirePred operating at a FPR of 20.0%.

Two variants introduce single-amino acid substitutions in the FHA domain of Chk2. The variant c.300G>T replaces Gln100 by a His residue. Although not frequent, this variant has been reported in the Genome Aggregation Database. Other replacements described at this position (Q100P, Q100R, and Q100K) are classified by ClinVar, gnomAD, or Ensembl as of uncertain significance. The sequence conservation of the position carrying the amino acid replacement is only moderate among Chk2 similar sequences. PirePred classifies the variant as a VUS, and the rMD analysis indicates that the variant is unlikely to induce structural destabilization. It appears not to detrimentally affect the stability of the FHA domain, its dimerization, or the catalytic center, but, as the cellular binding partners of Chk2 are not well-characterized, whether the variant can exert an effect on Chk2 interactions cannot be assessed at present. After the bioinformatics analysis, this variant remains a VUS. The second variant found in the FHA domain is c.503C>T, which causes replacement of The168 by Ile. This variant is located at a highly conserved position, and it has been reported in ClinVar and gnomAD as of uncertain significance and in Ensembl as of uncertain significance/likely pathogenic. Other replacements described at this position (T168N, T168P, and T168A) are classified by ClinVar as of uncertain significance, and one of them (T168N) is classified by Ensembl as of uncertain significance/likely pathogenic. PirePred classifies the variant as pathogenic, and the rMD analysis indicates this variation reduces the conformational stability of the protein. The conserved Thr residue is predicted not to be phosphorylated, so loss of a phosphorylation spot seems to be not related to its pathogenicity Thus, this variant seems pathogenic, the likely cause of it being a structural destabilization of the FHA domain.

Five variants have been found in the protein kinase domain. The variant c.844C>G leads to the replacement of His282 by aspartic acid, and it is not reported in gnomAD or in the 1000 Genomes Project. Other replacements described at this position (H282Q, H282R, H282N, and H282Y) are classified by ClinVar, gnomAD, or Ensembl as of uncertain significance. Its sequence conservation is high, and PirePred classifies the variant as pathogenic. The rMD simulations showed that this protein variant is stable, and therefore, the cause of pathogenicity would not be a structural destabilization of Chk2. Interestingly, this variant is annotated in VarSome as affecting the splicing, which could explain its pathogenicity. The variant c.1033C>T leads to the replacement of His345 by tyrosine. It is reported in ClinVar and gnomAD as of uncertain significance. Other replacements found at this position (H345Q, H345P, H345R, H345L, and H345D) are classified by ClinVar or gnomAD as of uncertain significance. The variant displays high sequence conservation. PirePred classifies it as pathogenic, and the rMD simulations show that this variant destabilizes the kinase domain. In addition, the replaced His residue forms an H-bond with the catalytic residue Asp347. This variant, in addition to destabilizing the structure, may detrimentally affect the catalytic activity. The variant c.1175C>T replaces Ala392 with valine. It has been reported in gnomAD with a low allele frequency of 6.4 × 10^−5^. ClinVar and gnomAD describe it as of uncertain significance. Other replacements found at this position (A392G, A392T, A392P, and A392E) are classified by ClinVar, gnomAD, or Ensembl as of uncertain significance. Its sequence conservation is high. PirePred classifies it as pathogenic, and the rMD data point to structural destabilization as a likely cause. It should be indicated that the amino acid residue replaced in this variant (Ala392) packs with Pro471 and Arg474 of the other kinase domain (the partner monomer in the dimer) and that variants affecting the Pro471 and Arg474 residues have also been found among the patients analyzed in this study (see below and Tables 1–3). The variant c.1412C>T replaces Pro471 with leucine. ClinVar describes it as of uncertain significance, and it is not reported in gnomAD or the 1000 Genomes Project. Other replacements found at this position (P471T and P471S) are classified by ClinVar or Ensembl as of uncertain significance. It displays high sequence conservation. PirePred classifies it as pathogenic, and rMD analysis points to structural destabilization as a cause. This variant replaces Pro471, which, as indicated above, is in contact with Ala392 (see discussion on the previous variant) and Arg474 (see discussion on the next variant). It appears that the interfaces between the two kinase domains, where Ala392, Pro471, and Arg474 are brought into contact, are hotspots where single-amino acid substitutions arising from single-nucleotide variations in *CHEK2* may cause pathogenicity. Finally, the variant c.1420C>T replaces Arg474 with Cys, and it has been reported at a low allele frequency both in the 1000 Genomes Project (1.7 × 10^−4^) and the gnomAD database (1.0 × 10^−5^). In ClinVar and gnomAD, it is reported as having conflicting interpretations of pathogenicity. Other replacements found at this position (R474L, R474G, and R474M) are classified by ClinVar or Ensembl as of uncertain significance; R474S is classified by ClinVar and gnomAD as of uncertain significance and by Ensembl as of uncertain significance/likely benign; and R474H is classified by ClinVar and gnomAD as having conflicting interpretations of pathogenicity. It displays high sequence conservation and is classified by PirePred as pathogenic, a likely cause being structural destabilization, as indicated by rMD simulations. Arg474 is in contact with Ala392 and Pro471 (see previous two variants), likely helping the FHA domain to strengthen the Chk2 interaction at the dimeric interface. rMD analyses also showed that replacements brought about in these three positions could affect Chk2 dimerization, thus being an additional cause for deleteriousness.

In conclusion, some *CHEK2* mutations have been associated with an increased risk for BC. Since the frequency of carriers may vary depending on the population, and different mutations may be associated with different cancer risks, more studies—in different populations—are needed to establish a complete range of risks associated with *CHEK2* founder alleles and to estimate the appropriated risks related to several mutations for different cancer sites. Notwithstanding, our in-depth bioinformatics analysis provides supporting evidence (Richards et al., 2015) for the pathogenic characteristics of six of the seven variants reported here. In addition, incorporation of MD analysis into the study of the variants at the protein level allowed us to discriminate between possible causes of pathogenicity. For most of the variants analyzed here, structural destabilization of either the FHA or the kinase domain appears to be a likely cause.

## Data Availability

The original contributions presented in the study are publicly available. This data is deposited in the ClinVar NCBI Repository (https://www.ncbi.nlm.nih.gov/clinvar/). Accession numbers: VCV000128059; VCV000141136; VCV000182427; VCV000220465; VCV000410053; VCV002691812; VCV002691813.
