# Supplementary material for: Association between missense variants of uncertain significance in the CHEK2 gene and hereditary breast cancer: a cosegregation and bioinformatics analysis
Source: Front Genet. 2024 Feb 27;14:1274108. doi: 10.3389/fgene.2023.1274108 (PMC10927753; doi:10.3389/fgene.2023.1274108)
Supplement: Supplementary file 1 [file DataSheet1.PDF]

SUPPORTING INFORMATION for

Association between Missense Variants of Uncertain Significance in *CHEK2* Gene and Hereditary Breast Cancer: A Bioinformatic Analysis.

Content

SI Methods

Mutation Modeling, Relaxation Molecular Dynamics Setup and Trajectory Analyses.

SI Tables

Table S1.

SI Figures

Figures S1-S14.

SI References

SI Methods

Mutation Modeling, Relaxation Molecular Dynamics Setup and Trajectory Analyses. Mutations were modeled on the wild-type structures through Swiss-PdbViewer v4.1.0(1) by replacing the wild type residue side chain by that present in the VUS variant, selecting the conformer of lowest energy that showed no clashes. To run the simulations, systems were prepared in truncated dodecahedral boxes (1nm-minimum distance between protein atoms and the box edges) filled with Tip3p water molecules, and the force field Charmm22 with CMAP correction (version 2.0) (2) was set. Default protonation states associated to a neutral pH were set along with a salt concentration (Na^+ and Cl^- ions) of 0.154 M, which mimics the ionic strength of a physiological saline solution (approximately isotonic to blood serum). A short steepest descent (3) minimization step was first launched (20,000 steps of 1 fs) on the solvated systems. Minimized systems of each protein domain (FHA and kinase-like) were then triplicated (3 replicas) at each of the three temperatures (T) simulated (**Table S1**). To select the working temperatures a series of simulations was prior carried out over an extended range of high temperatures (higher than the protein mid denaturation temperature) (not shown). The objective was finding a suitable temperature interval where the mutated proteins showed a differential behavior (prior destabilization) compared to wild-type at the time that the unfolding process is accelerated, enabling the saving of computing power and time. Replicated systems were heated through a heating ladder that enabled to gradually increase the temperature by 50 K (every 50 ps, using a timestep of 1 fs) until reaching the working temperature. An NVT ensemble was settled through the v-rescale thermostat (4)for each of these partial heating steps. Then, two serial NPT equilibration steps were followed by setting in both the v-rescale thermostat, and as barostats Berendsen for the first step with 1 atm, whereas for the second one Parrinello-Rahman(5) with the same pressure. The first of these equilibration steps ran by 250 ps with restraints imposed on the heavy atoms, whereas the second one ran during 500 ps without any restraint. The Verlet cutoff-scheme algorithm(6,7) was settled for accounting van der Waals interactions and the PME method (8) for electrostatic interactions, both with a cutoff value of 1 nm (10 Å). The 1µs-productive phase was collected under the same conditions (**Table S1**), and simulations and trajectory analyses were run with GROMACS 2020 package(9)

Moreover, the ADP molecule left in the catalytic site along rMDs was parameterized as follow: first, its coordinates were extracted from the crystal structure (2CN5)(10), and the partial atomic charges modeled with Gaussian 09 (11) (HF/6-31G*). Then, the charges were fitted through the RESP method (12,13) (implemented in Antechamber20 (14,15) and parameters obtained from the General Amber Force Field (GAFF)(16) through LEaP, a program of Antechamber20 suite(15,16).

For stability analysis, conformational local (1nm-radius around the center of mass of the mutated residue) unstability was followed through an *ad hoc* conformational clustering (agglomerative algorithm as implemented in the scikit-learn Python package (17).with a linkage distance cutoff of 0.2 nm, which based on two dimensional root-mean-square deviation (2D-RMSD) matrices relating frames of a wild-type reference trajectory to frames of a mutant trajectory. To obtain a 2D-RMSD matrix a metatrajectory is first built from a wild-type trajectory (reference) and a mutant one (target). The global behaviour (conformational stability) of the protein along the simulated trajectories was also investigated through a battery of trajectory analyses, e.g. radius of gyration, secondary structure content, TM-score, RMSF (Root Mean Square Fluctuation), SASA (Solvent Accessible Surface Area), H-bonds (intra-protein and protein/water), which were comparatively done (wild-type *versus* mutant). In the case of the kinase domain, which is simulated as a dimer, the local clustering is performed on each chain individually. More details on the analyses carried out are given along the figures caption and legends provided in this **Supporting Material**.

Table S1. rMD setup broken down by simulation steps.[a]

Simulation Step	General Settings (cutoffs)	PBC & Constraints	Step Setting	Thermodynamic Ensemble (baths)	Phys. Conditions	Sim. Time
Minimization	Integrator: steepest descent, Neighboring searching: grid, rcoul (PME): 1.0 nm, rvdw (cut-off): 1.0 nm	PBC: xyz, Constraints: none	Emtol: 1.0 kJ/mol	-	Temp: 0 K Press: 0 atm	max 20,000 steps
Heating	Integrator: md, Neighboring searching: grid, rcoul (PME): 1.0 nm, rvdw (cut-off): 1.0 nm, vdw-modifier: potential-shift-verlet	PBC: xyz, Constraints: h-bonds	t-step: 1 fs	NVT, Termost: Berendsen, Barost: no	Temp: Ini-T + ramp (n x 50 K) Press: 0 atm	n x 50 ps
Equilibration 1			t-step: 2 fs	NPT, Termost: v-rescale, Barost: Berendsen	Temp: Final-T[b] Press: 1 atm	250 ps
Equilibration 2			t-step: 2 fs	NPT, Termost: v-rescale, Barost: Parrinello-Rahman	Temp: Final-T[b] Press: 1 atm	500 ps
Production						1 µs
						**Total:** 1.01 µs

[a] MD setup settled with Gromacs 2020 package (9)

[b] Working temperatures were selected as described in Methods of the main text. For rMD simulations of FHA domain (simulated as a monomer) final temperatures tested were 398, 418 and 438 K, while for kinase domain (simulated as a dimer) they were 358, 378 and 398 K.

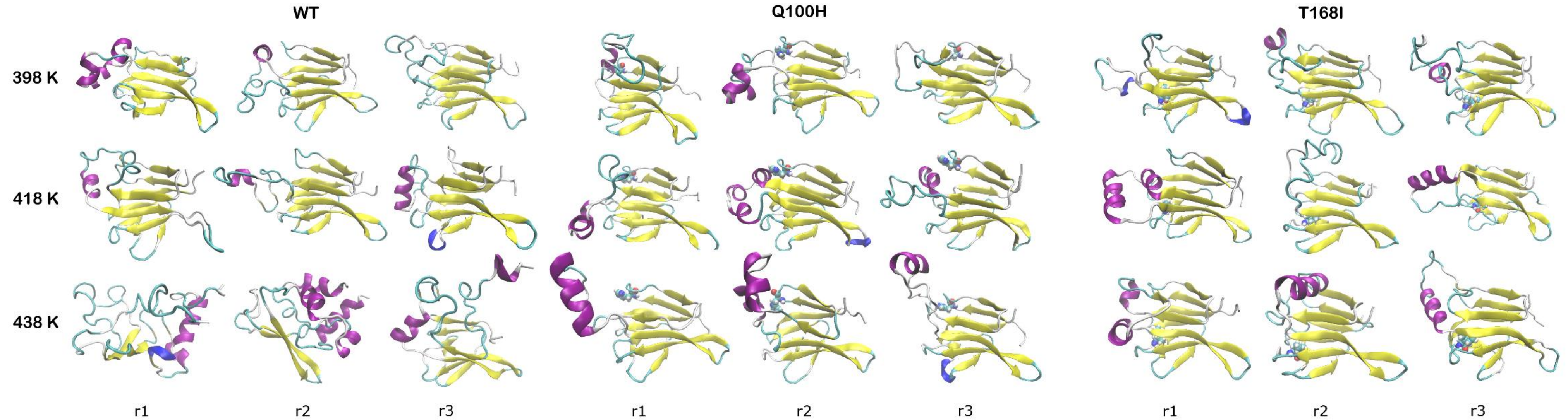

Figure S1. Final structures after 1 µs rMD simulation of β-sandwich FHA domain (WT) and the two mutants thereof (Q100H and T168I). Conformations obtained at the final frame (1 µs) of trajectory for each replica run. The flexible long loop encompassing residues 116-143 appears always oriented into the left. Mutated residue in Q100H and T168I are depicted in spheres with Nitrogen atoms colored in blue, Carbon in cyan and Oxygen in red. β-sandwich core is retained in all the variants at the three temperatures except in WT FHA at 438K, where it appears partially or completely destroyed.

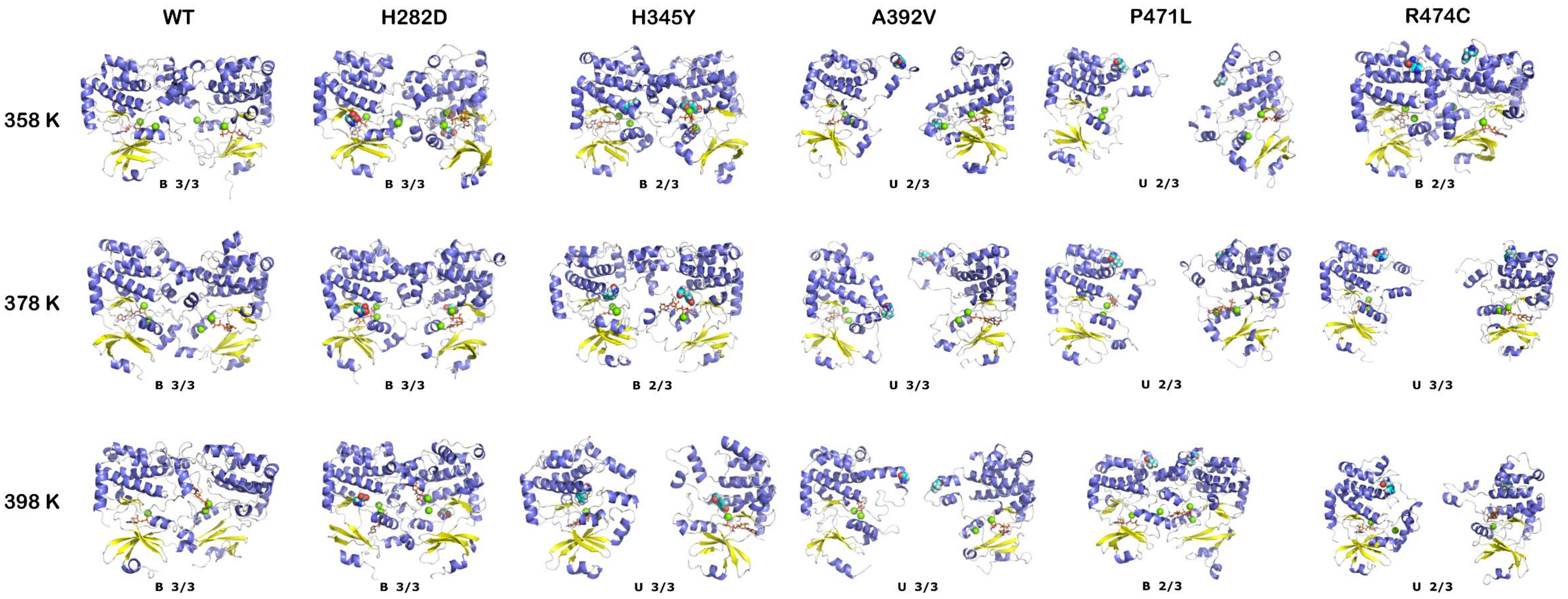

Figure S2. Final structure and dimerization state after 1 μs rMD simulation of kinase domain (WT) and mutants thereof. For each temperature (row) and mutant (column) the most prevalent dimerization state (bound (B) or unbound (U) with the fraction of replicas, below every structure) obtained at the end of the rMD trajectory (final frame, 1 μs) is represented by one out of the final conformations among those showing the predominant state. In the case of the unbound (U) conformations, chains have been unnaturally displaced (after separation they appear in random relative positions in the collected final frame) to appear approximately in the same disposition (face to face) and distance between them in order to further show the effect of the mutation on the global stability of the individual chains. Mutated residues are depicted in spheres with Nitrogen atoms colored in blue, Carbon in cyan and Oxygen in red. Mg^{2+} ions (spheres in green) and ADP substrate (sticks in orange) in the catalytic site are also shown. In a few cases, a Mg^{2+} and/or an ADP moved out into the bulk from their original positions in the crystal, so that they do not appear represented in the structure.

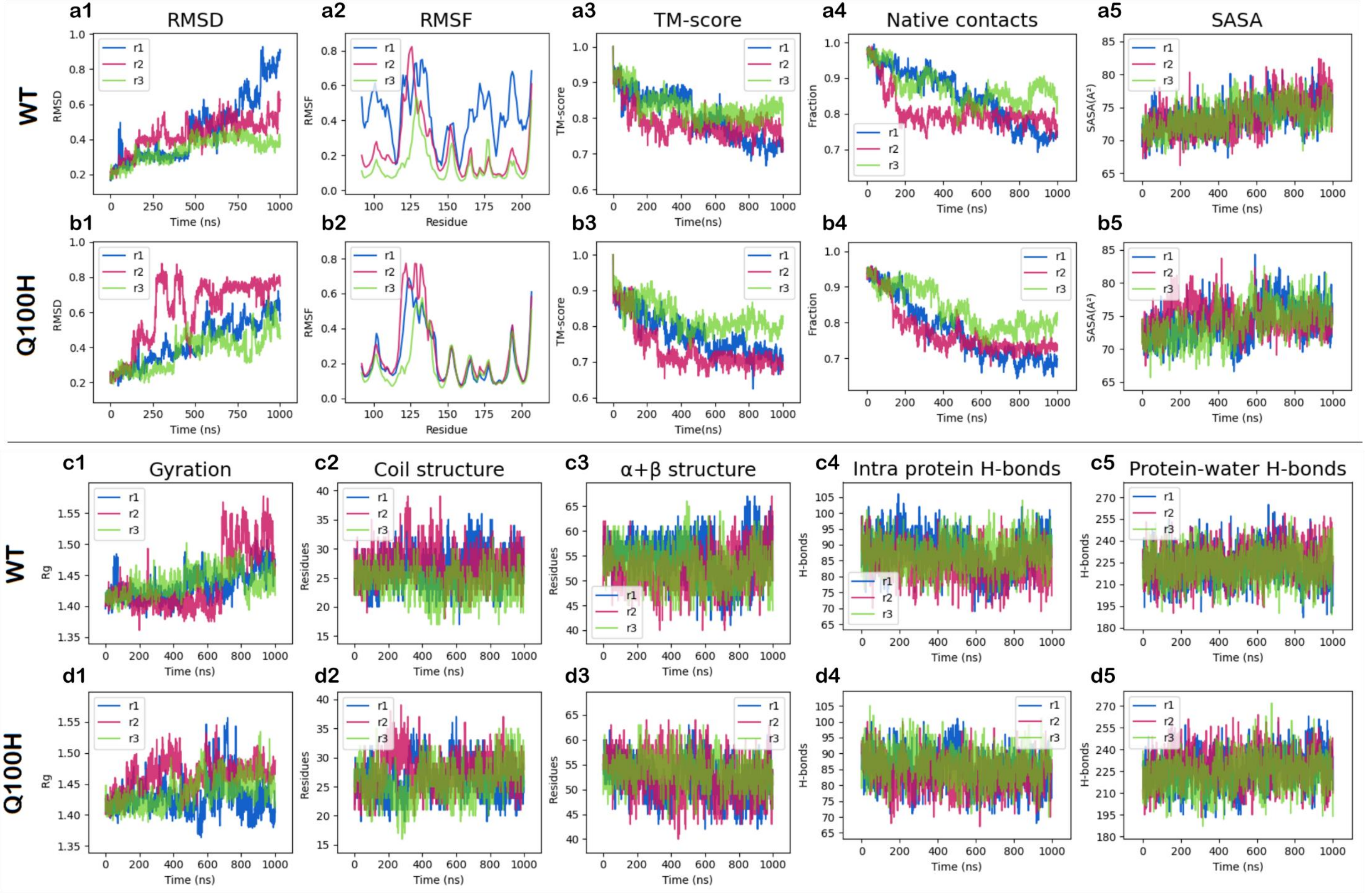

WT
Q100H
a1
RMSD
a2
RMSF
a3
TM-score
a4
Native contacts
a5
SASA
b1
b2
b3
b4
b5
c1
Gyration
c2
Coil structure
c3
α+β structure
c4
Intra protein H-bonds
c5
Protein-water H-bonds
d1
d2
d3
d4
d5
r1
r2
r3
RMSD
RMSF
TM-score
Fraction
SASA(A²)
Rg
Residues
H-bonds
Time (ns)
Time(ns)
Residue

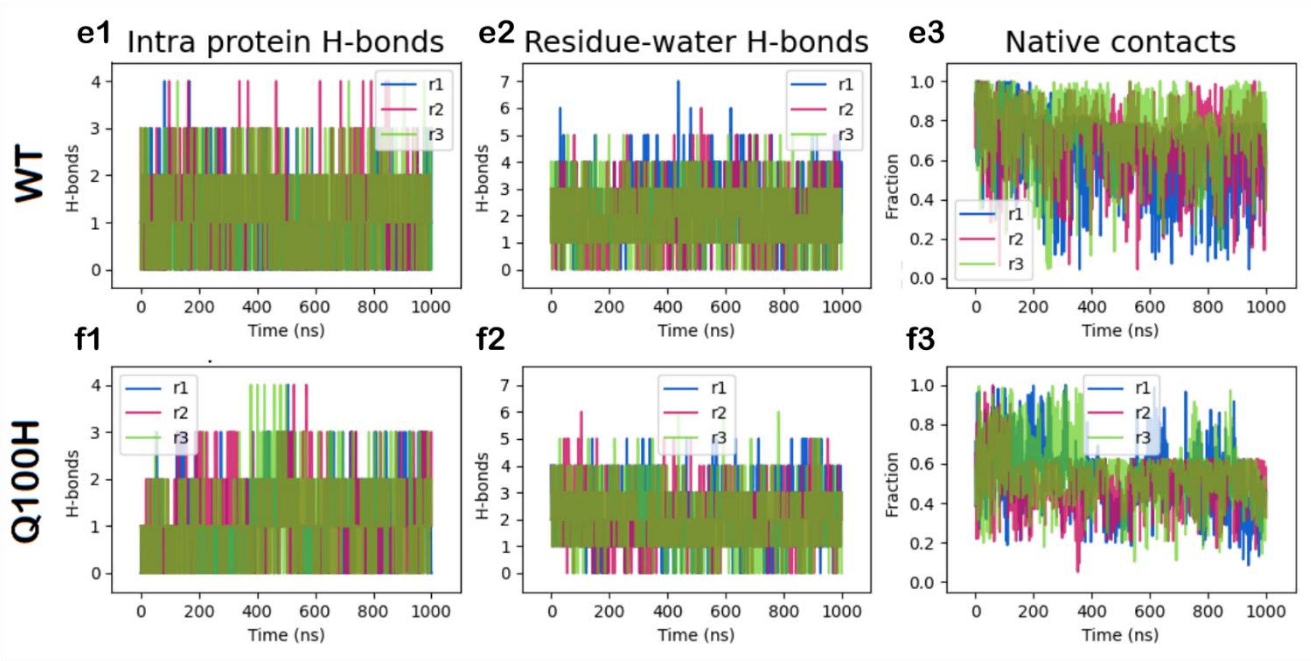

Figure S3. Plots of parameters assessed on productive rMD trajectories of mutant Gln100His (FHA domain) compared to WT protein, at 398 K. The results for the WT protein are represented in the **a**, **c** and **e** rows, while the variant is represented in rows **b**, **d** and **f**. **a1)** and **b1)** Root Mean Square Deviation (RMSD) of the backbone of the protein along the rMD simulation, using the first frame as reference. **a2)** and **b2)** Root Mean Square Fluctuation (RMSF) of the backbone of the protein along the rMD simulation per residue. **a3)** and **b3)** TM-score of the backbone of the protein along the rMD simulation, using the first frame as reference. **a4)** and **b4)** Global (for the whole protein) native contacts of the backbone along the rMD simulation, using the first frame as reference. **a5)** and **b5)** Global Solvent Accessible Surface Area (SASA) of the protein along the rMD simulation, using the first frame as reference. **c1)** and **d1)** Radius of gyration of the backbone of the protein along the rMD simulation. **c2)** and **d2)** Number of residues of the protein in random coil secondary structure (according to DSSP (18) along the rMD simulation. **c3)** and **d3)** Number of residues of the protein in α-helix or β-sheet secondary structure (according to DSSP (18) along the rMD simulation. **c4)** and **d4)** Global number of hydrogen bonds between the backbone of pairs of residues in the protein along the rMD simulation. **c5)** and **d5)** Global number of hydrogen bonds between the backbone of a residue in the protein and a water molecule along the rMD simulation. **e1)** and **f1)** Local number of hydrogen bonds between the backbone of the affected residue and the rest of the protein (backbone) along the rMD simulation. **e2)** and **f2)** Local number of hydrogen bonds between the backbone of the affected residue and water molecules along the rMD simulation. **e3)** and **f3)** Local native contacts established by the affected residue (backbone) along the rMD simulation, using the first frame as reference. In **e1-3)** and **f1-3)** interactions are considered to be local if one of the interacting partners (protein residues or water molecules) is the affected residue for this variant (Q100 in the WT protein, H100 in the variant).

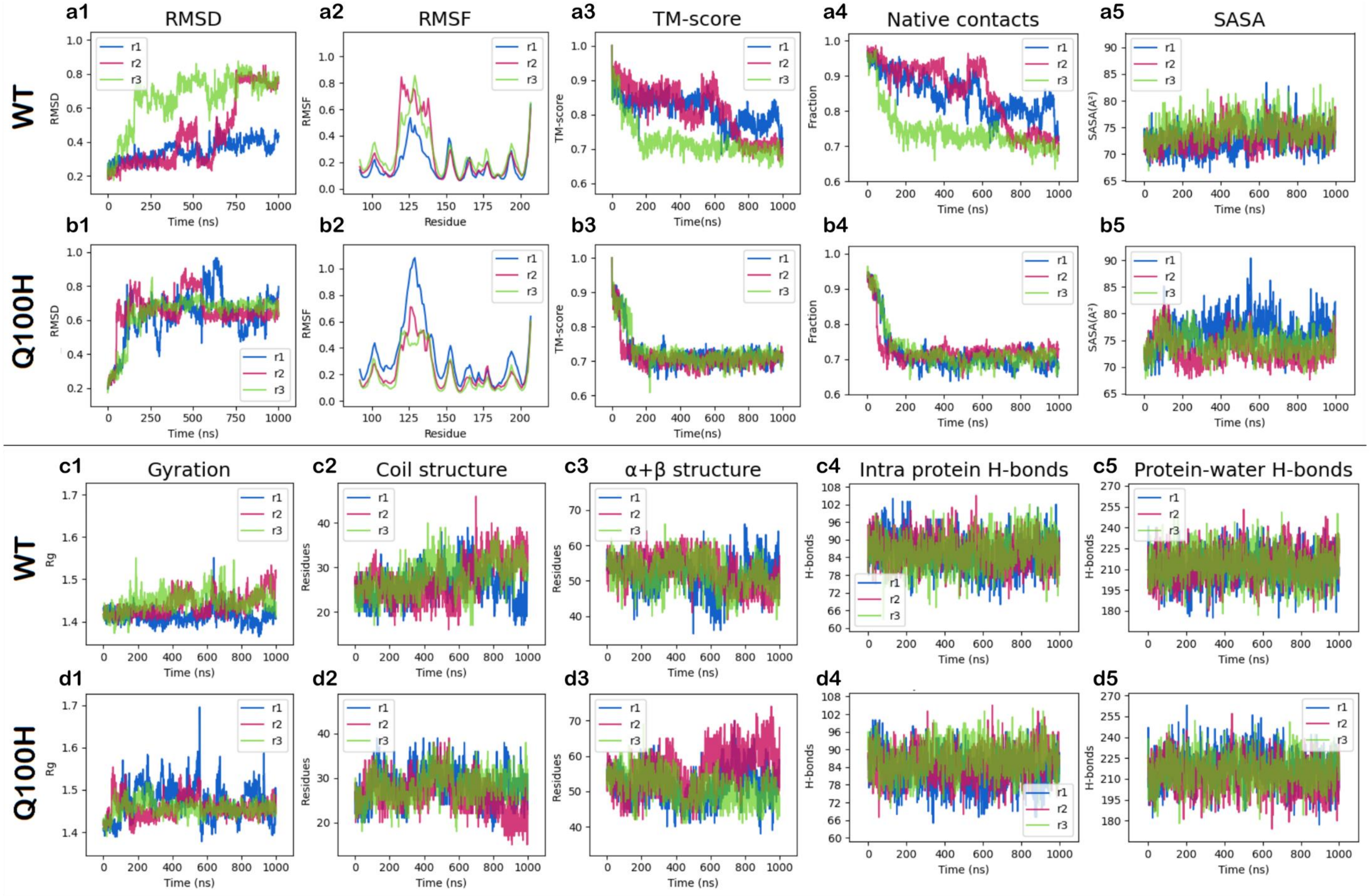
WT
Q100H
a1 RMSD
a2 RMSF
a3 TM-score
a4 Native contacts
a5 SASA
b1
b2
b3
b4
b5
c1 Gyration
c2 Coil structure
c3 α+β structure
c4 Intra protein H-bonds
c5 Protein-water H-bonds
d1
d2
d3
d4
d5
r1
r2
r3
RMSD
RMSF
TM-score
Fraction
SASA(A²)
Rg
Residues
H-bonds
Time (ns)
Time(ns)
Residue

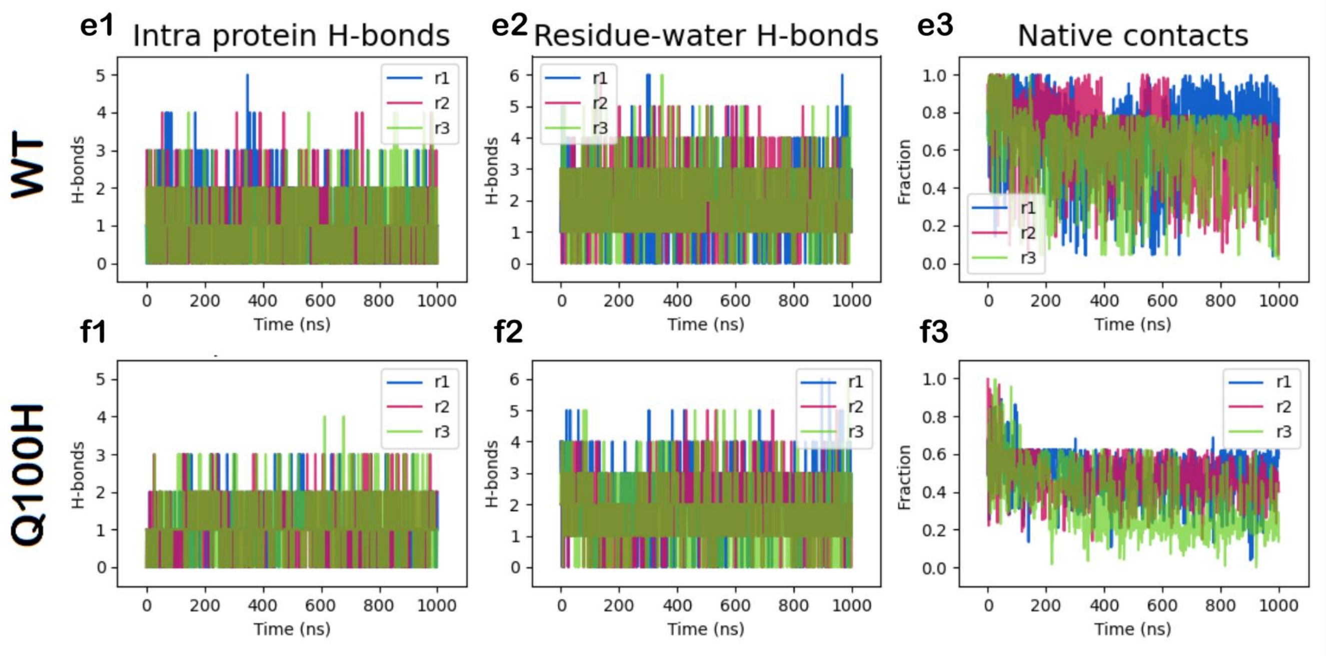

Figure S4. Plots of parameters assessed on rMD trajectories of mutant Gln100His (FHA domain) compared to WT protein, at 418 K. The results for the WT protein are represented in the **a**, **c** and **e** rows, while the variant is represented in rows **b**, **d** and **f**. **a1)** and **b1)** Root Mean Square Deviation (RMSD) of the backbone of the protein along the rMD simulation, using the first frame as reference. **a2)** and **b2)** Root Mean Square Fluctuation (RMSF) of the backbone of the protein along the rMD simulation per residue. **a3)** and **b3)** TM-score of the backbone of the protein along the rMD simulation, using the first frame as reference. **a4)** and **b4)** Global (for the whole protein) native contacts of the backbone along the rMD simulation, using the first frame as reference. **a5)** and **b5)** Global Solvent Accessible Surface Area (SASA) of the protein along the rMD simulation, using the first frame as reference. **c1)** and **d1)** Radius of gyration of the backbone of the protein along the rMD simulation. **c2)** and **d2)** Number of residues of the protein in random coil secondary structure (according to DSSP (18)long the rMD simulation. **c3)** and **d3)** Number of residues of the protein in α-helix or β-sheet secondary structure (according to DSSP (18)along the rMD simulation. **c4)** and **d4)** Global number of hydrogen bonds between the backbone of pairs of residues in the protein along the rMD simulation. **c5)** and **d5)** Global number of hydrogen bonds between the backbone of a residue in the protein and a water molecule along the rMD simulation. **e1)** and **f1)** Local number of hydrogen bonds between the backbone of the affected residue and the rest of the protein (backbone) along the rMD simulation. **e2)** and **f2)** Local number of hydrogen bonds between the backbone of the affected residue and water molecules along the rMD simulation. **e3)** and **f3)** Local native contacts established by the affected residue (backbone) along the rMD simulation, using the first frame as reference. In **e1-3)** and **f1-3)** interactions are considered to be local if one of the interacting partners (protein residues or water molecules) is the affected residue for this variant (Q100 in the WT protein, H100 in the variant).

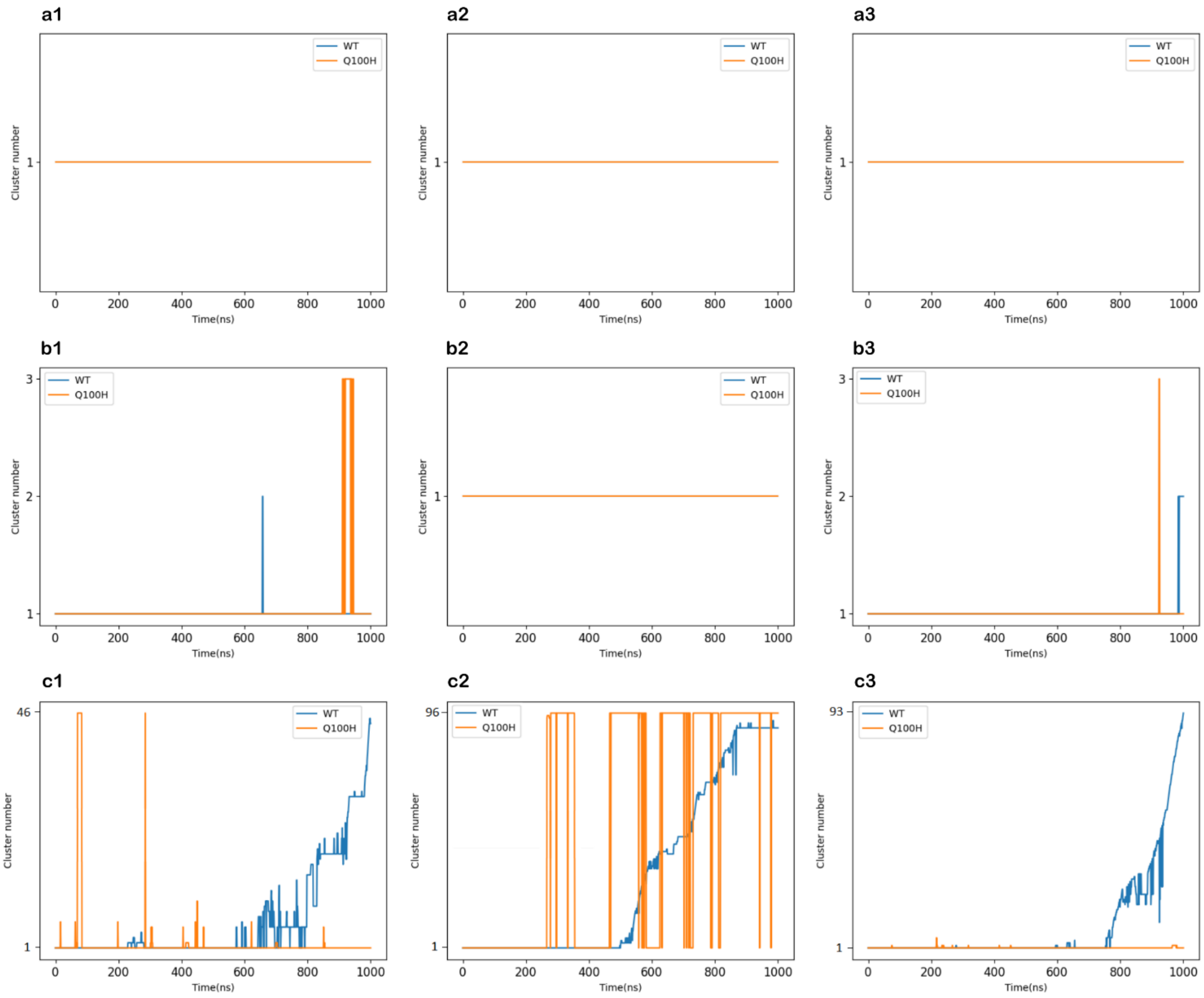

Figure S5. Plots of the comparative 2D-RMSD-based local clustering (0.3nm-distance cutoff) performed on rMD trajectories of mutant Gln100His (FHA domain) versus WT protein. a1-a3) Clustering plots of rMDs at 398 K comparing a1) replica 1 of WT versus replica 1 of Q100H, a2) replica 2 of WT versus replica 2 of Q100H, and a3) replica 3 of WT versus replica 3 of Q100H. **b1-b3)** Clustering plots of rMDs at 418 K depicting comparatively the same replicas pairs as in a) plots. **c1-c3)** Clustering plots of rMDs at 438 K depicting comparatively the same replicas pairs as in a) and b) plots. Color-coding indicated in the legends.

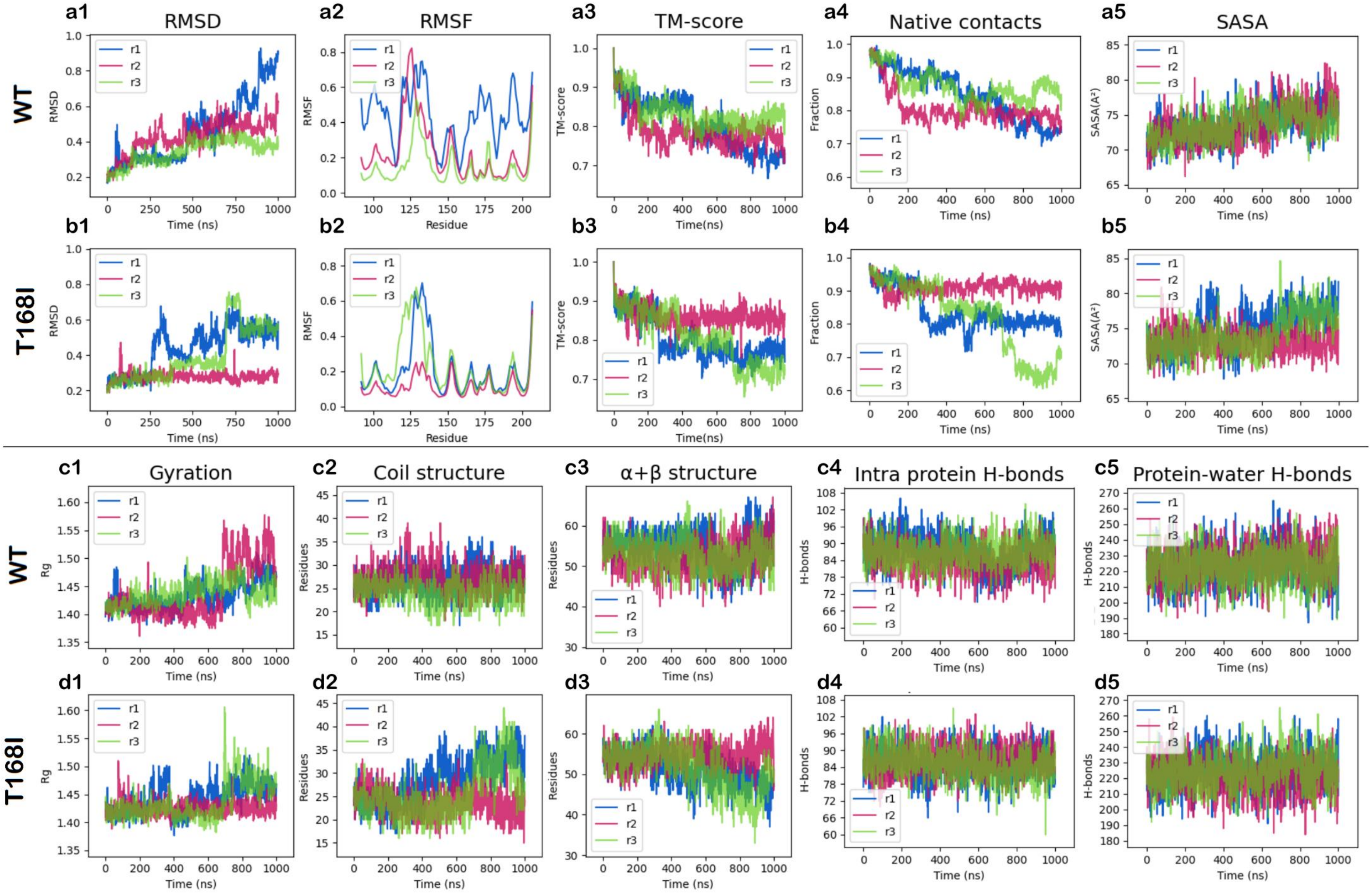
WT
T168I
a1 RMSD
a2 RMSF
a3 TM-score
a4 Native contacts
a5 SASA
b1
b2
b3
b4
b5
c1 Gyration
c2 Coil structure
c3 α+β structure
c4 Intra protein H-bonds
c5 Protein-water H-bonds
d1
d2
d3
d4
d5
RMSD
RMSF
TM-score
Fraction
SASA(A²)
Rg
Residues
H-bonds
Time (ns)
Time(ns)
Residue
r1
r2
r3

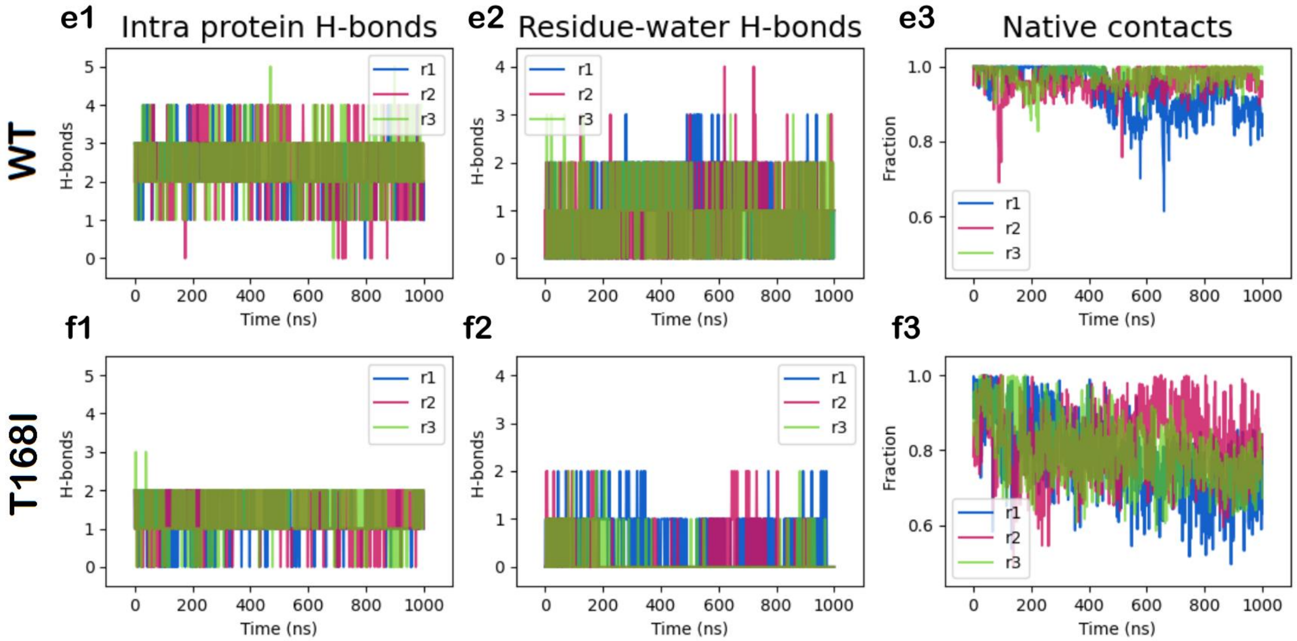

Figure S6. Plots of parameters assessed on rMD trajectories of mutant Thr168Ile (FHA domain) compared to WT protein, at 398 K. The results for the WT protein are represented in the **a**, **c** and **e** rows, while the variant is represented in rows **b**, **d** and **f**. **a1)** and **b1)** Root Mean Square Deviation (RMSD) of the backbone of the protein along the rMD simulation, using the first frame as reference. **a2)** and **b2)** Root Mean Square Fluctuation (RMSF) of the backbone of the protein along the rMD simulation per residue. **a3)** and **b3)** TM-score of the backbone of the protein along the rMD simulation, using the first frame as reference. **a4)** and **b4)** Global (for the whole protein) native contacts of the backbone along the rMD simulation, using the first frame as reference. **a5)** and **b5)** Global Solvent Accessible Surface Area (SASA) of the protein along the rMD simulation, using the first frame as reference. **c1)** and **d1)** Radius of gyration of the backbone of the protein along the rMD simulation. **c2)** and **d2)** Number of residues of the protein in random coil secondary structure (according to DSSP (18)along the rMD simulation. **c3)** and **d3)** Number of residues of the protein in α-helix or β-sheet secondary structure (according to DSSP (18)long the rMD simulation. **c4)** and **d4)** Global number of hydrogen bonds between the backbone of pairs of residues in the protein along the rMD simulation. **c5)** and **d5)** Global number of hydrogen bonds between the backbone of a residue in the protein and a water molecule along the rMD simulation. **e1)** and **f1)** Local number of hydrogen bonds between the backbone of the affected residue and the rest of the protein (backbone) along the rMD simulation. **e2)** and **f2)** Local number of hydrogen bonds between the backbone of the affected residue and water molecules along the rMD simulation. **e3)** and **f3)** Local native contacts established by the affected residue (backbone) along the rMD simulation, using the first frame as reference. In **e1-3)** and **f1-3)** interactions are considered to be local if one of the interacting partners (protein residues or water molecules) is the affected residue for this variant (T168 in the WT protein, I168 in the variant).

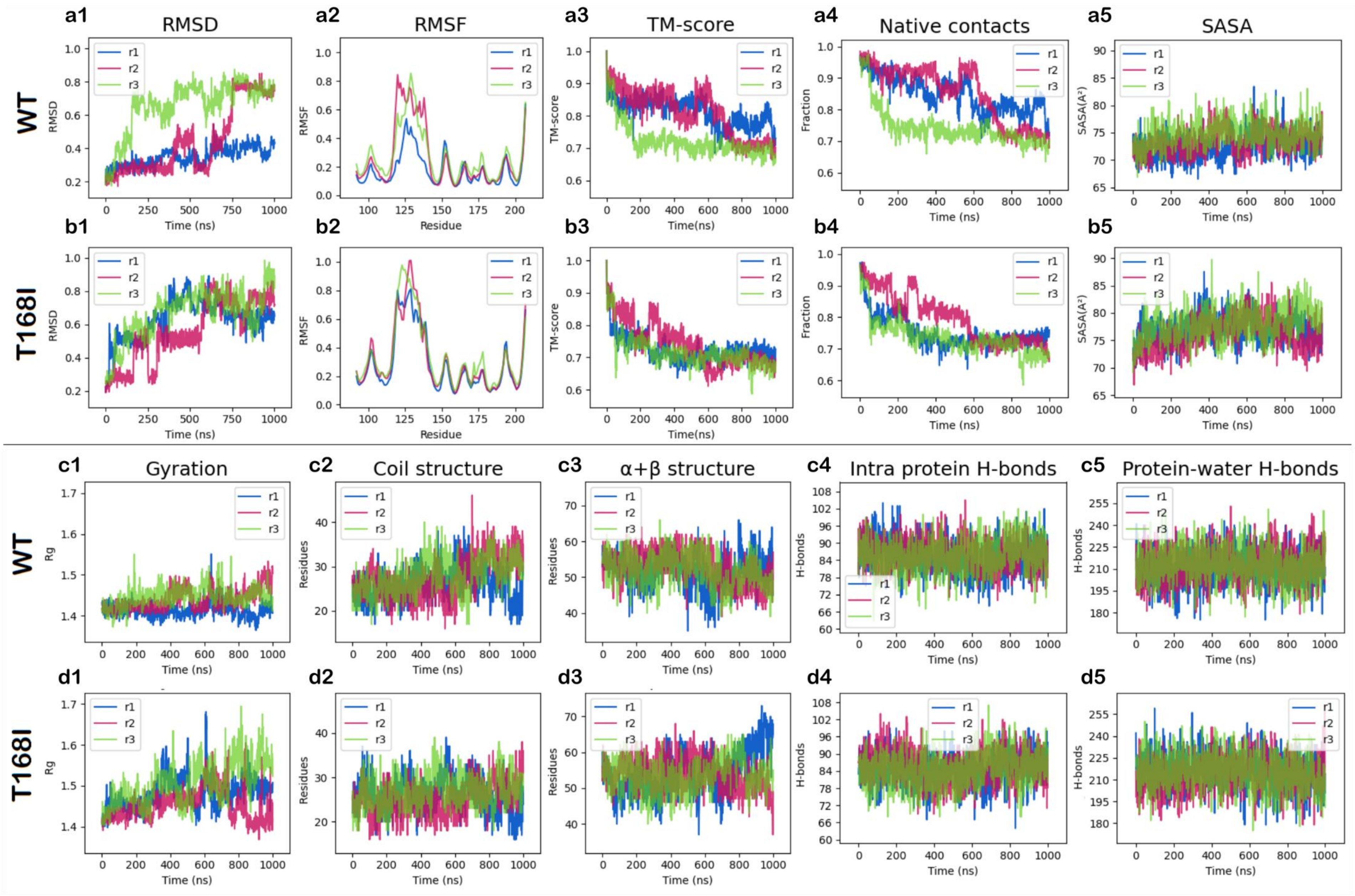
a1
RMSD
a2
RMSF
a3
TM-score
a4
Native contacts
a5
SASA
WT
b1
b2
b3
b4
b5
T168I
RMSD
RMSF
TM-score
Fraction
SASA(A²)
Time (ns)
Residue
Time(ns)
r1
r2
r3
c1
Gyration
c2
Coil structure
c3
α+β structure
c4
Intra protein H-bonds
c5
Protein-water H-bonds
d1
d2
d3
d4
d5
Rg
Residues
H-bonds

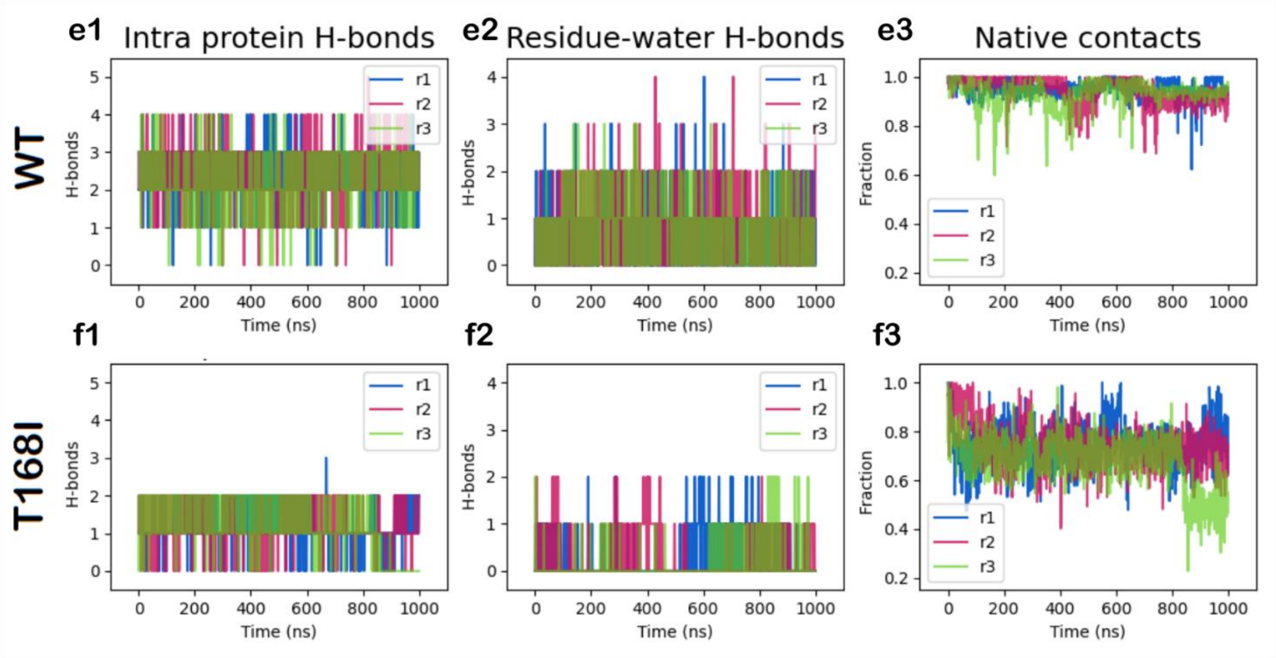

Figure S7. Plots of parameters assessed on rMD trajectories of mutant Thr168Ile (FHA domain) compared to WT protein, at 418 K. The results for the WT protein are represented in the **a**, **c** and **e** rows, while the variant is represented in rows **b**, **d** and **f**. **a1)** and **b1)** Root Mean Square Deviation (RMSD) of the backbone of the protein along the rMD simulation, using the first frame as reference. **a2)** and **b2)** Root Mean Square Fluctuation (RMSF) of the backbone of the protein along the rMD simulation per residue. **a3)** and **b3)** TM-score of the backbone of the protein along the rMD simulation, using the first frame as reference. **a4)** and **b4)** Global (for the whole protein) native contacts of the backbone along the rMD simulation, using the first frame as reference. **a5)** and **b5)** Global Solvent Accessible Surface Area (SASA) of the protein along the rMD simulation, using the first frame as reference. **c1)** and **d1)** Radius of gyration of the backbone of the protein along the rMD simulation. **c2)** and **d2)** Number of residues of the protein in random coil secondary structure (according to DSSP(18)along the rMD simulation. **c3)** and **d3)** Number of residues of the protein in α-helix or β-sheet secondary structure (according to DSSP (18)long the rMD simulation. **c4)** and **d4)** Global number of hydrogen bonds between the backbone of pairs of residues in the protein along the rMD simulation. **c5)** and **d5)** Global number of hydrogen bonds between the backbone of a residue in the protein and a water molecule along the rMD simulation. **e1)** and **f1)** Local number of hydrogen bonds between the backbone of the affected residue and the rest of the protein (backbone) along the rMD simulation. **e2)** and **f2)** Local number of hydrogen bonds between the backbone of the affected residue and water molecules along the rMD simulation. **e3)** and **f3)** Local native contacts established by the affected residue (backbone) along the rMD simulation, using the first frame as reference. In **e1-3)** and **f1-3)** interactions are considered to be local if one of the interacting partners (protein residues or water molecules) is the affected residue for this variant (T168 in the WT protein, I168 in the variant).

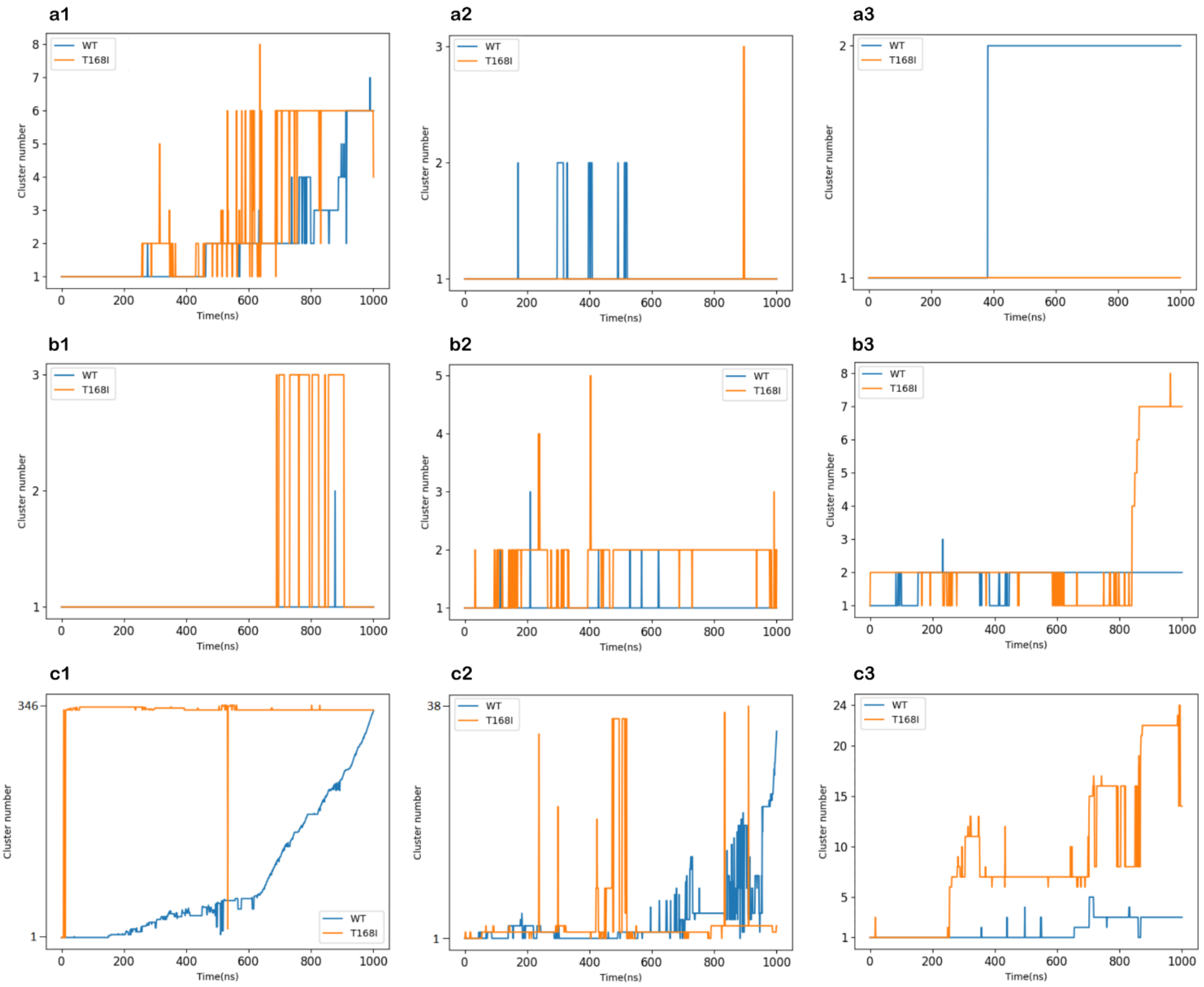

Figure S8. Plots of the comparative 2D-RMSD-based local clustering (0.3nm-distance cutoff) performed on rMD trajectories of mutant Thr168Ile (FHA domain) versus WT protein. a1-a3) Clustering plots of rMDs at 398 K comparing a1) replica 1 of WT versus replica 1 of T168I, a2) replica 2 of WT versus replica 2 of T168I, and a3) replica 3 of WT versus replica 3 of T168I. **b1-b3)** Clustering plots of rMDs at 418 K depicting comparatively the same replicas pairs as in a) plots. **c1-c3)** Clustering plots of rMDs at 438 K depicting comparatively the same replicas pairs as in a) and b) plots. Color-coding indicated in the legends.

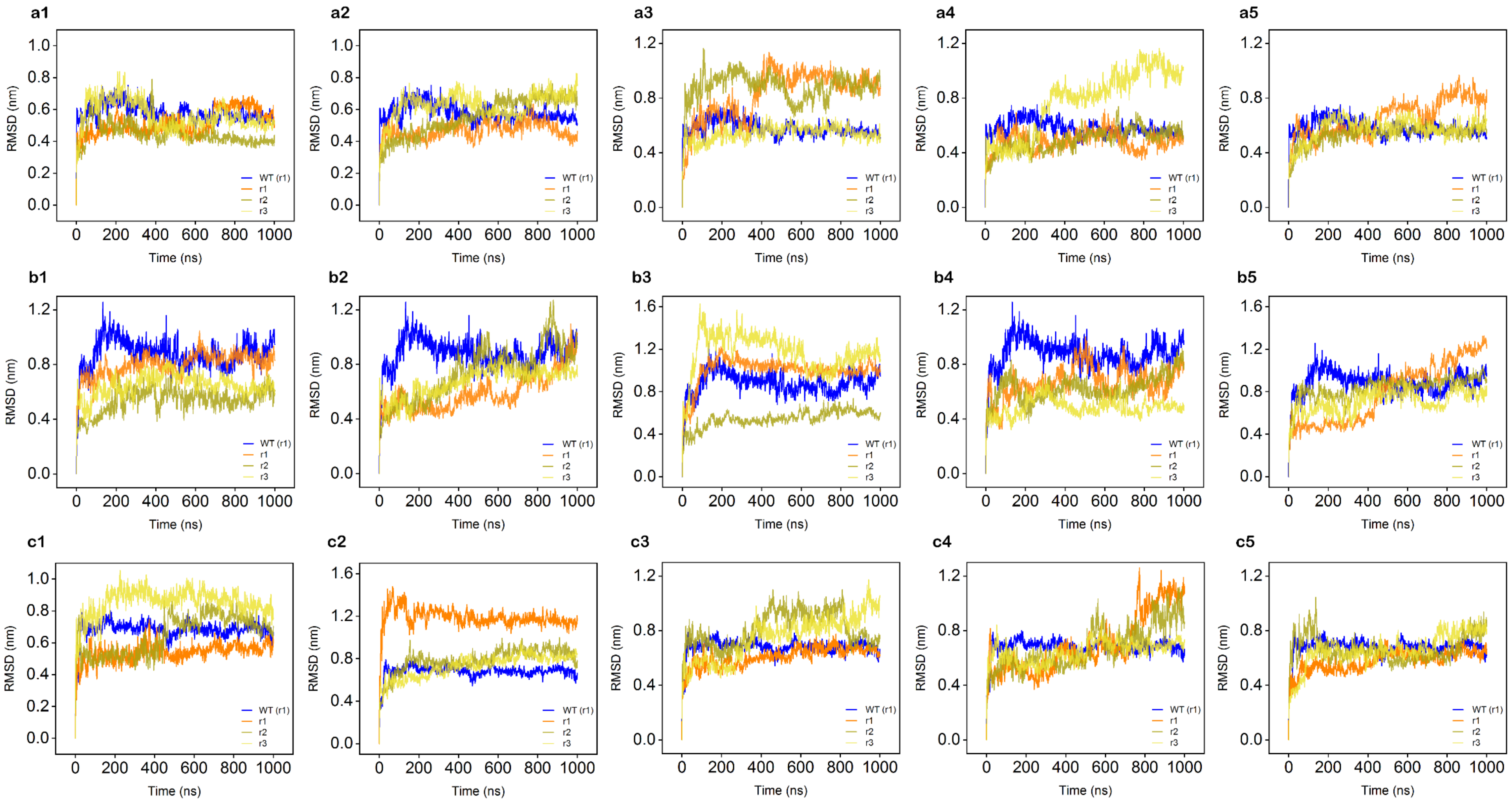

Figure S9. RMSD comparison between kinase domain wild-type (replica 1) and mutants along rMD trajectories. RMSD (backbone) versus the minimized structure (see **SI Methods** and **Table S1**) for WT kinase domain and the VUS variants detected in BC patients in this study. **a1-a5)** Depict comparatively the RMSD plots obtained for replica 1 of WT kinase domain (blue line) and the 3 replicas of VUS variants 1) H282D, 2) H345Y, 3) A392V, 4) P471L and 5) R474C simulated at 358 K. **b1-b5)** Idem. to plots in a) now for simulations run at 378 K. **c1-c5)** Idem. to plots in a) now for simulations run at 398 K.

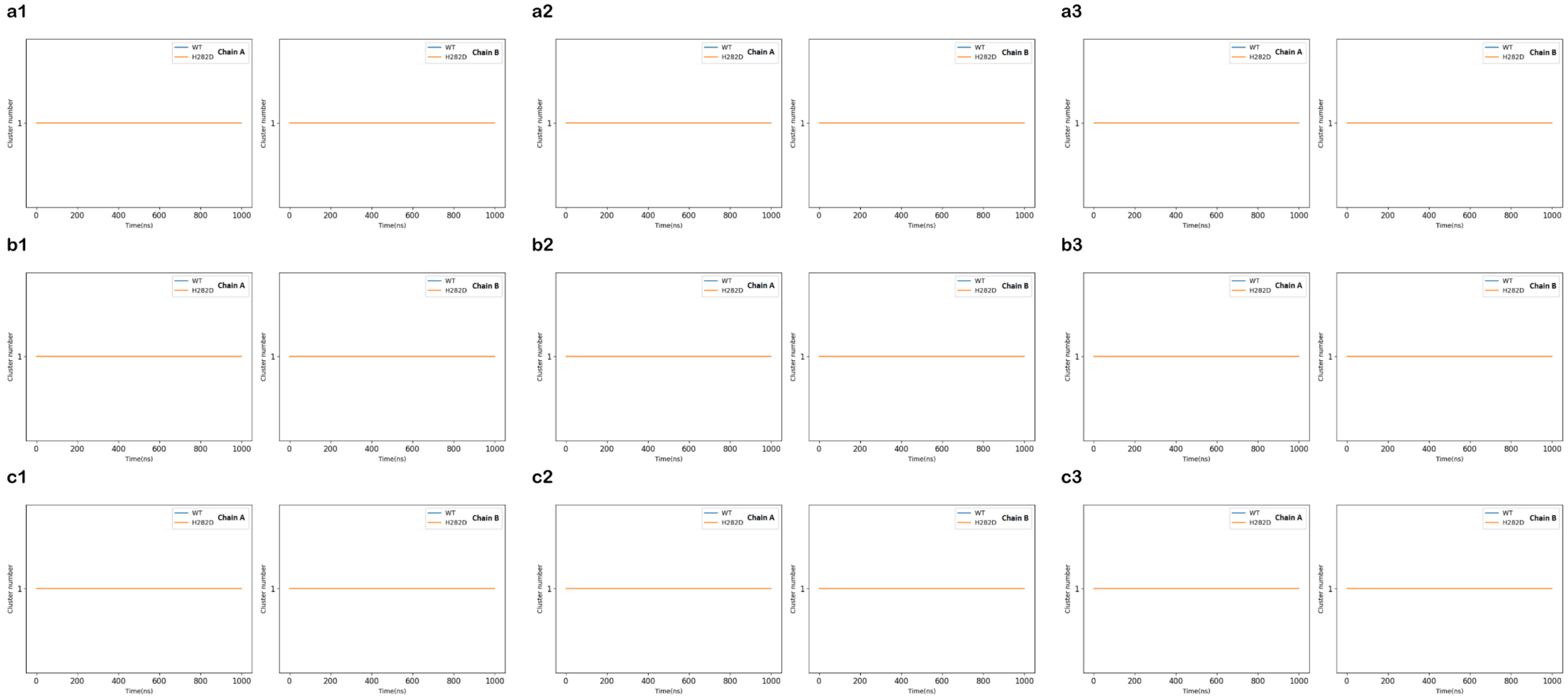

Figure S10. Plots of the comparative 2D-RMSD-based local clustering (0.3nm-distance cutoff) performed on rMD trajectories of mutant His282Asp (kinase domain) versus WT protein. a1-a3) Clustering plots of rMDs at 358 K comparing a1) chain A in replica 1 of WT versus chain A in replica 1 of H282D (left), and chain B in replica 1 of WT versus chain B in replica 1 of H282D (right), a2) chain A in replica 2 of WT versus chain A in replica 2 of H282D (left), and chain B in replica 2 of WT versus chain B in replica 2 of H282D (right), and a3) chain A in replica 3 of WT versus chain A in replica 3 of H282D (left), and chain B in replica 3 of WT versus chain B in replica 3 of H282D (right). **b1-b3)** Clustering plots of rMDs at 378 K depicting comparatively the same chains and replicas pairs as in a) plots. **c1-c3)** Clustering plots of rMDs at 398 K depicting comparatively the same chains and replicas pairs as in a) and b) plots. Color-coding indicated in the legends.

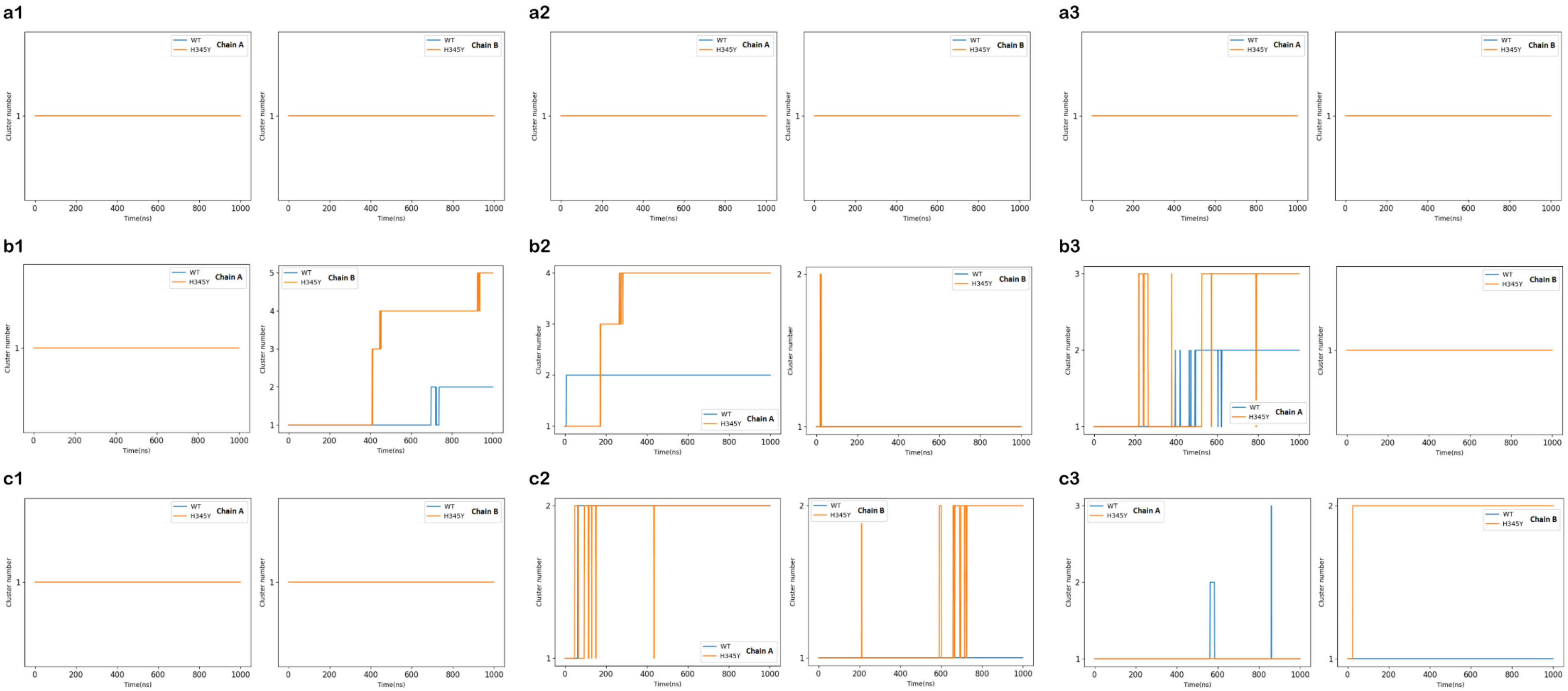

Figure S11. Plots of the comparative 2D-RMSD-based local clustering (0.3nm-distance cutoff) performed on rMD trajectories of mutant His345Tyr (kinase domain) versus WT protein. a1-a3) Clustering plots of rMDs at 358 K comparing a1) chain A in replica 1 of WT versus chain A in replica 1 of H345Y (left), and chain B in replica 1 of WT versus chain B in replica 1 of H345Y (right), a2) chain A in replica 2 of WT versus chain A in replica 2 of H345Y (left), and chain B in replica 2 of WT versus chain B in replica 2 of H345Y (right), and a3) chain A in replica 3 of WT versus chain A in replica 3 of H345Y (left), and chain B in replica 3 of WT versus chain B in replica 3 of H345Y (right). **b1-b3)** Clustering plots of rMDs at 378 K depicting comparatively the same chains and replicas pairs as in a) plots. **c1-c3)** Clustering plots of rMDs at 398 K depicting comparatively the same chains and replicas pairs as in a) and b) plots. Color-coding indicated in the legends.

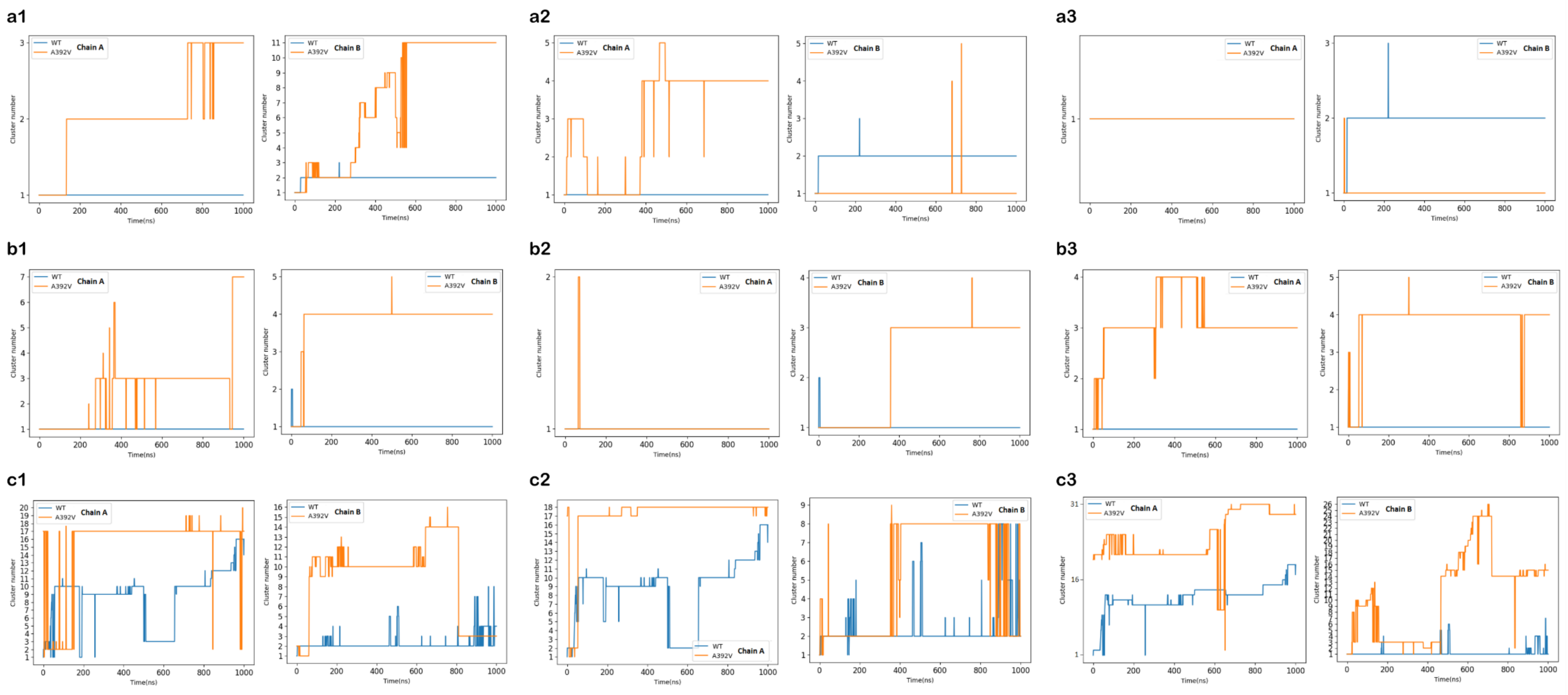

Figure S12. Plots of the comparative 2D-RMSD-based local clustering (0.3nm-distance cutoff) performed on rMD trajectories of mutant Ala392Val (kinase domain) versus WT protein. a1-a3) Clustering plots of rMDs at 358 K comparing a1) chain A in replica 1 of WT versus chain A in replica 1 of A392V (left), and chain B in replica 1 of WT versus chain B in replica 1 of A392V (right), a2) chain A in replica 2 of WT versus chain A in replica 2 of A392V (left), and chain B in replica 2 of WT versus chain B in replica 2 of A392V (right), and a3) chain A in replica 3 of WT versus chain A in replica 3 of A392V (left), and chain B in replica 3 of WT versus chain B in replica 3 of A392V (right). **b1-b3)** Clustering plots of rMDs at 378 K depicting comparatively the same chains and replicas pairs as in a) plots. **c1-c3)** Clustering plots of rMDs at 398 K depicting comparatively the same chains and replicas pairs as in a) and b) plots. Color-coding indicated in the legends.

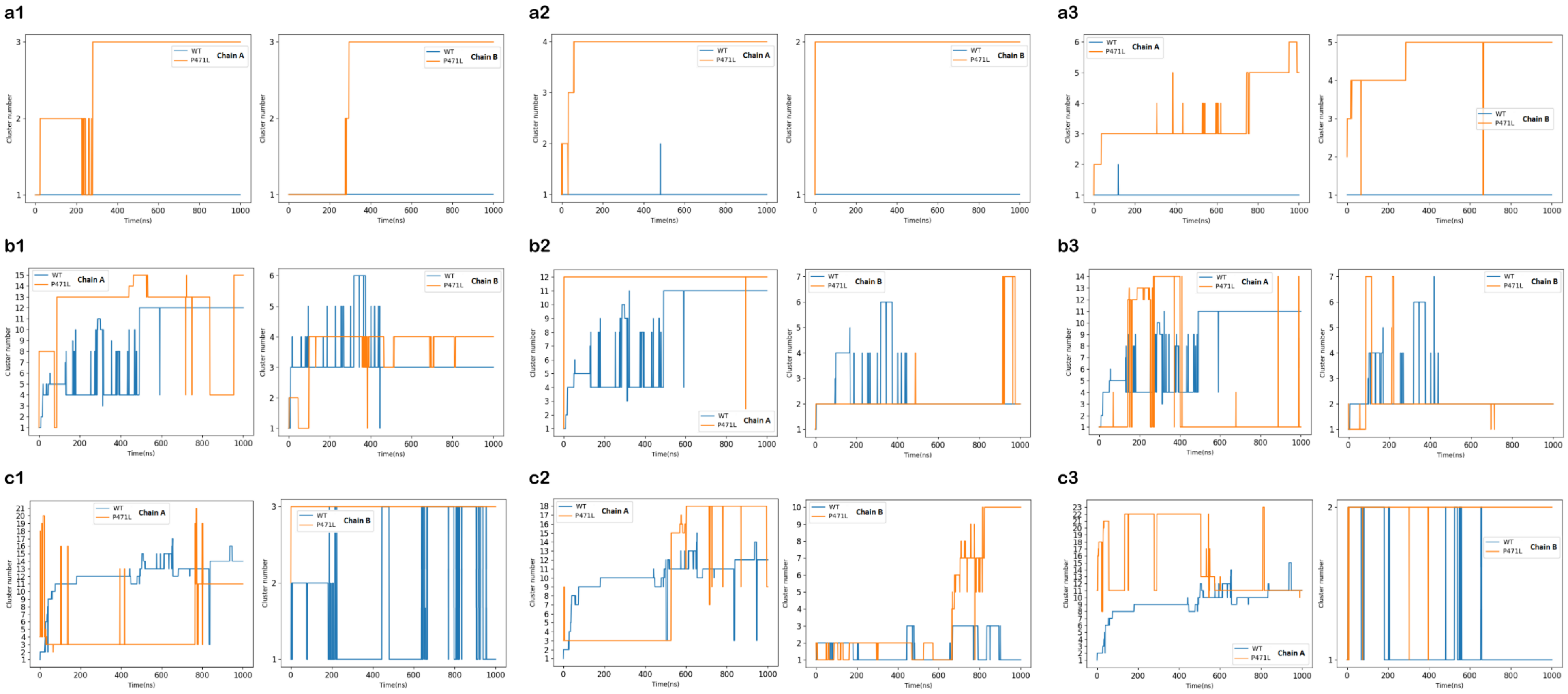

Figure S13. Plots of the comparative 2D-RMSD-based local clustering (0.3nm-distance cutoff) performed on rMD trajectories of mutant Pro471Leu (kinase domain) versus WT protein. a1-a3) Clustering plots of rMDs at 358 K comparing a1) chain A in replica 1 of WT versus chain A in replica 1 of P471L (left), and chain B in replica 1 of WT versus chain B in replica 1 of P471L (right), a2) chain A in replica 2 of WT versus chain A in replica 2 of P471L (left), and chain B in replica 2 of WT versus chain B in replica 2 of P471L (right), and a3) chain A in replica 3 of WT versus chain A in replica 3 of P471L (left), and chain B in replica 3 of WT versus chain B in replica 3 of P471L (right). **b1-b3)** Clustering plots of rMDs at 378 K depicting comparatively the same chains and replicas pairs as in a) plots. **c1-c3)** Clustering plots of rMDs at 398 K depicting comparatively the same chains and replicas pairs as in a) and b) plots. Color-coding indicated in the legends.

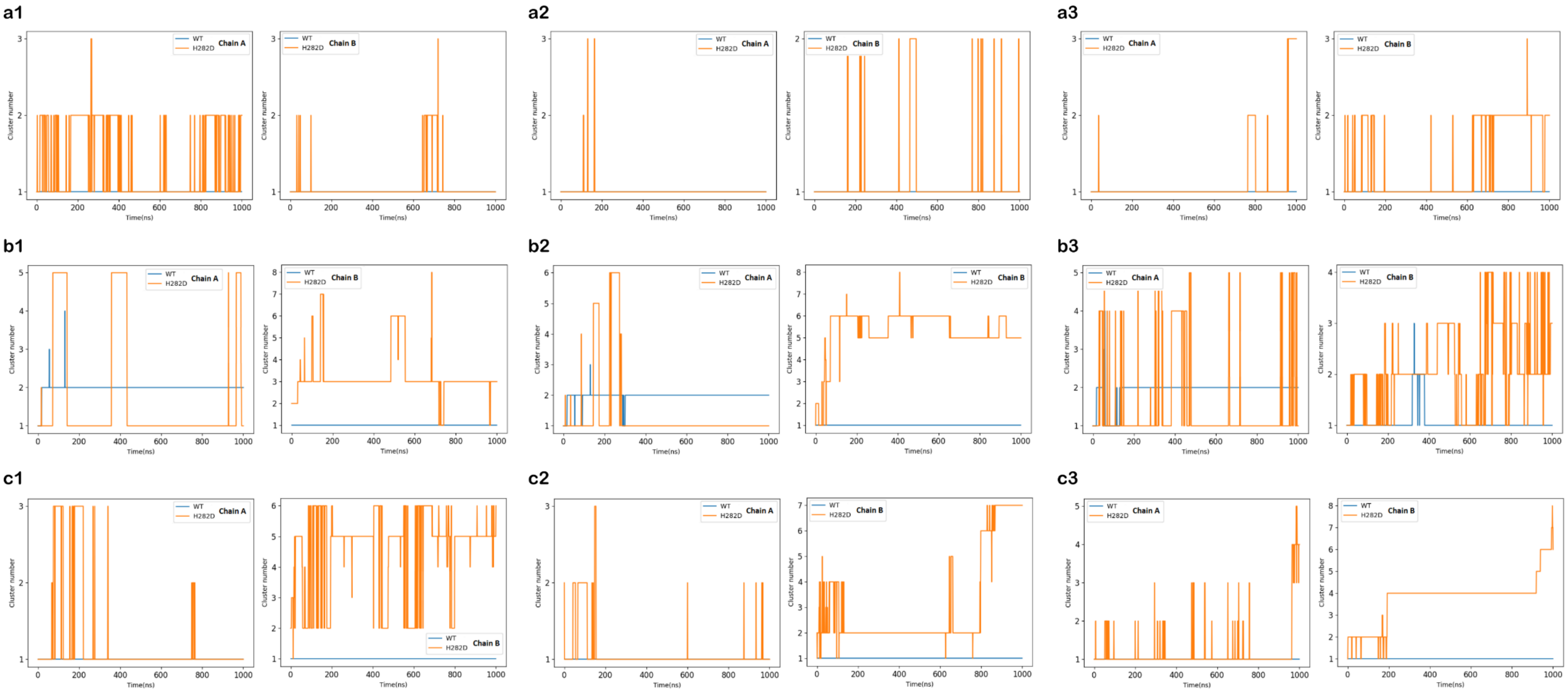

Figure S14. Plots of the comparative 2D-RMSD-based local clustering (0.3nm-distance cutoff) performed on rMD trajectories of mutant Arg474Cys (kinase domain) versus WT protein. a1-a3) Clustering plots of rMDs at 358 K comparing a1) chain A in replica 1 of WT versus chain A in replica 1 of R474R (left), and chain B in replica 1 of WT versus chain B in replica 1 of R474R (right), a2) chain A in replica 2 of WT versus chain A in replica 2 of R474R (left), and chain B in replica 2 of WT versus chain B in replica 2 of R474R (right), and a3) chain A in replica 3 of WT versus chain A in replica 3 of R474R (left), and chain B in replica 3 of WT versus chain B in replica 3 of R474R (right). **b1-b3)** Clustering plots of rMDs at 378 K depicting comparatively the same chains and replicas pairs as in a) plots. **c1-c3)** Clustering plots of rMDs at 398 K depicting comparatively the same chains and replicas pairs as in a) and b) plots. Color-coding indicated in the legends.

SI References

1. Guex, N, Peitsch MC. SWISS-MODEL and the Swiss-PdbViewer: an environment for comparative protein modeling. Electrophoresis. 1997;18(15):2714-23.

2. Mackerell AD, Feig M, Brooks CL. Extending the treatment of backbone energetics in protein force fields: Limitations of gas-phase quantum mechanics in reproducing protein conformational distributions in molecular dynamics simulation. J Comput Chem. 2004;25(11):1400-15.

3. Haug EJ, Arora JS, Matsui K. A steepest-descent method for optimization of mechanical systems. J Optim Theory Appl.1976;19(3).

4. Bussi G, Donadio D, Parrinello M. Canonical sampling through velocity rescaling. J Chem Phys. 2007;126(1):014101.

5. Parrinello M, Rahman A. Polymorphic transitions in single crystals: A new molecular dynamics method. J Appl Phys. 1981;52(12).

6. Verlet L. Computer "experiments" on classical fluids. I. Thermodynamical properties of Lennard-Jones molecules. Phys Rev.1967;159(1).

7. Páll S, Hess B. A flexible algorithm for calculating pair interactions on SIMD architectures. Computer Phys Commun. 2013;184(12).

8. Essmann U, Perera L, Berkowitz ML, Darden T, Lee H, Pedersen LG. A smooth particle mesh Ewald method. J Chem Phys. 1995;103(19).

9. Van Der Spoel D, Lindahl E, Hess B, Groenhof G, Mark AE, Berendsen HJC. GROMACS: Fast, flexible, and free. J Comput Chem. 2005:26(16):1701-18.

10. Oliver AW, Paul A, Boxall KJ, Barrie SE, Aherne GW, Garrett MD, et al. Trans-activation of the DNA-damage signalling protein kinase Chk2 by T-loop exchange. EMBO J. 2006;25(13):3179-90.

11. Frisch MJ, Trucks GW, Schlegel HB, Scuseria GE, Robb MA, Cheeseman JR, et al. Gaussian 09, Revision A. 02; Gaussian, Inc: Wallingford, CT, 2016..

12. Cieplak P, Cornell WD, Bayly C, Kollman PA. Application of the multimolecule and multiconformational RESP methodology to biopolymers: Charge derivation for DNA, RNA, and proteins. J Comput Chem.1995;16(11).

13. Bayly CI, Cieplak P, Cornell WD, Kollman PA. A well-behaved electrostatic potential based method using charge restraints for deriving atomic charges: The RESP model. J Phys Chem. 1993;97(40).

14. Case DA, Belfon K, Ben-Shalom IY, Brozell SR, Cerutti DS, Cheatham TE, et al.AMBER 2020.University of California.

15. Wang J, Wang W, Kollman PA, Case DA. Automatic atom type and bond type perception in molecular mechanical calculations. J Mol Graph Model. 2006;25(2):247-60.

16. Wang J, Wolf RM, Caldwell JW, Kollman PA, Case DA. Development and testing of a general Amber force field. J Comput Chem. 2004;25(9):1157-74.

17. Pedregosa F, Varoquaux G, Gramfort A, Michel V, Thirion B, Grisel O, et al. Scikit-learn: Machine learning in Python. J Mach Learn Res 2011;12.

18. Kabsch W, Sander C. Dictionary of Protein Secondary Structure: Pattern Recognition of Hydrogen-Bonded and Geometrical Features. Biopolymers 1983;22(12):2577-637.
